# TRAMFIX: TRavelling Across Melbourne for FIXel-based analysis (a reproducibility and reliability study)

**DOI:** 10.1162/IMAG.a.1153

**Published:** 2026-03-10

**Authors:** Remika Mito, Sila Genc, Jocelyn Halim, Joseph Yuan-Mou Yang, Jacques-Donald Tournier, Michael Kean, Chris Kokkinos, Richard McIntyre, Maria A. Di Biase, Robert E. Smith, Andrew Zalesky

**Affiliations:** Department of Psychiatry, The University of Melbourne, Parkville, VIC, Australia; Florey Department of Neuroscience and Mental Health, The University of Melbourne, Parkville, VIC, Australia; Developmental Imaging, Clinical Sciences, Murdoch Children’s Research Institute, Parkville, VIC, Australia; Neuroscience Advanced Clinical Imaging Service (NACIS), Department of Neurosurgery, The Royal Children’s Hospital, Parkville, VIC, Australia; Neuroscience Research, Murdoch Children’s Research Institute, Parkville, VIC, Australia; Department of Paediatrics, The University of Melbourne, Parkville, VIC, Australia; Department of Early Life Imaging, School of Biomedical Engineering & Imaging Sciences, King’s College London, London, United Kingdom; Department of Medical Imaging, The Royal Children’s Hospital, Parkville, VIC, Australia; Florey Institute of Neuroscience and Mental Health, Heidelberg, VIC, Australia; Monash Biomedical Imaging, Monash University, Clayton, VIC, Australia; Department of Anatomy and Physiology, The University of Melbourne, Melbourne, VIC, Australia; Department of Biomedical Engineering, The University of Melbourne, Parkville, VIC, Australia

**Keywords:** diffusion MRI, fixel-based analysis, reproducibility, reliability

## Abstract

Fixel-based analysis (FBA) has gained substantial interest for its ability to probe fibre-specific changes in the brain’s white matter from diffusion-weighted imaging data. However, the reproducibility and reliability of fixel-based measures across different scanners remains largely unknown. In this work, we present TRAMFIX (TRavelling Across Melbourne for FIXel-based analysis): a multisite dataset of traveling participants (n = 10 healthy adults) scanned across four 3T MRI scanners using a harmonised multi-shell diffusion-weighted imaging (DWI) protocol. DWI data were processed using two pipelines that can be adopted when performing multi-site FBA studies (site-specific vs. pooled processing). We extracted fixel-based measures of fibre density (FD), fibre cross-section (FC), and fibre density and cross-section (FDC) from the harmonized protocol. While the primary goal was to assess reproducibility and reliability of FBA metrics, we additionally computed diffusion tensor imaging (DTI)-based fractional anisotropy (FA) and mean diffusivity (MD) for the purposes of comparison with previous studies. Within-subject coefficients of variation (CV_ws_) and intraclass correlation coefficients (ICC) of FBA and DTI measures were computed at multiple resolutions of computation and analysis: (i) the whole-brain averaged level, (ii) tract-level, and (iii) fixel- or voxel-level. Fixel-based metrics demonstrated high reproducibility and reliability at the whole-brain level (CV_ws_ ranging between 0.51% to 1.57% and ICC between 0.782 and 0.994). While reproducibility and reliability remained high for tract-averaged FBA measures (particularly the FC and FDC metrics), some tracts exhibited lower ICC values < 0.8 for the FD measure. When examining fixel-level reliability and reproducibility, clear spatial patterns emerged, with lower ICC across subcortical and cerebellar regions, and higher CV_ws_ at the cortical boundaries. FBA metrics demonstrated comparable, if not slightly better, reliability than tensor-based metrics derived from a subset of the same data. Our findings provide support for the reproducibility and reliability of fixel-based measures, highlighting their potential for use in multi-site FBA studies. Future work examining protocol-related differences, as well as appropriate harmonization strategies when pooling data across sites and scanners, will be valuable. To facilitate this, we provide the TRAMFIX dataset as a resource for investigating reproducibility, reliability, and harmonization of fixel-based analysis measures.

## 1 Introduction

Diffusion-weighted imaging (DWI; or diffusion MRI) is currently the only tool available to non-invasively map the human brain’s complex white matter architecture. While the development of diffusion tensor imaging (DTI) was a revolutionary step in our ability to quantify changes to the brain’s white matter connections (Basser et al., 1994), known limitations to the technique’s modeling assumptions and biological interpretations (Jones et al., 2013) have led to major improvements in DWI acquisition and modeling approaches. The past few decades have seen a rapid rise in the development and application of these advanced DWI techniques, enabling improved characterization of the brain’s complex white matter microstructure.

Fixel-Based Analysis (FBA; Raffelt et al., 2017) is one such framework that can provide more specific insight into the brain’s white matter fiber pathways. By modeling high-angular resolution diffusion imaging (HARDI) data with constrained spherical deconvolution (CSD; Dell’Acqua & Tournier, 2019), FBA can derive quantitative measures even in the presence of multiple fibre directions within voxels (known as ‘fixels’), enabling fibre-specific analysis of white matter pathways. The FBA framework has now been widely adopted across a range of clinical applications (see Dhollander et al. (2021) for a review), including in neurodegeneration (Ahmadi et al., 2024; Mito et al., 2018), psychopathology (Grazioplene et al., 2022; Kristensen et al., 2023, 2024; Lyon et al., 2019), and neurological disorders (Mito et al., 2022, 2024; Raffelt et al., 2017; Vaughan et al., 2017), as well as in healthy development (Genc et al., 2018; Genc, Malpas, et al., 2020) and aging (Kelley et al., 2021; Tinney et al., 2024). A key advantage of the technique is in its ability to identify fiber tract-specific changes, overcoming some of the key limitations to DTI and voxel-aggregate scalars (Mito et al., 2018; Raffelt et al., 2017).

Despite the technique’s growing popularity, FBA studies have thus far been largely limited to single-site designs. A key barrier for multi-site diffusion MRI studies more broadly is that quantitative measures are highly susceptible to differences between scanners and protocols, which can pose a major issue when pooling data across sites (Pinto et al., 2020). FBA poses additional challenges for data harmonization, as recommended pipelines necessitate the construction of study-specific templates, and CSD modeling approaches may differ based on acquisition (e.g., multi-shell vs. single-shell) (Dhollander & Connelly, 2016; Jeurissen et al., 2014). Although the extent of scanner- or protocol-related variability has been well documented for other DWI-based measures (Besseling et al., 2012; Cai et al., 2021; Fan et al., 2021; Grech-Sollars et al., 2015; Koller et al., 2021; Luque Laguna et al., 2020; Schilling et al., 2021; Vollmar et al., 2010), the extent to which site or scanner differences impact fixel-based measures is unclear.

The goal of this study was to assess the reproducibility and reliability of fixel-based measures using participants scanned across multiple sites (a ‘travelling heads’ study). Here, we present the TRAMFIX (TRavelling Across Melbourne for FIXel-based analysis) cohort, in which 10 healthy adults were scanned across four 3 T MRI scanners located at three different sites with an identical DWI protocol. We computed fixel-based metrics using two pipelines that could be used when combining data across sites (site-specific vs. pooled processing). Reproducibility and reliability of these measures across sites were assessed at three different levels of analysis: (i) averaged across the whole-brain; (ii) averaged across white matter tracts; and (iii) at the individual fixel-level. While the terms reproducibility and reliability are often used interchangeably, they represent different properties (Shoukri et al., 2008), and were assessed using within-subject coefficient of variation (CV_ws_) and intraclass correlation coefficient (ICC), respectively. For the purposes of comparison with previous DTI studies, we also examined the reproducibility and reliability of tensor-based metrics at these equivalent three levels (whole-brain, tract-averaged, and voxel-level).

## 2 Methods

### 2.1 Participants

Ten neurologically healthy adult volunteers (7 female, age range: 19–30 years) were recruited into the study via internal email advertisement and notice boards at participating institutions (University of Melbourne, the Florey Institute of Neuroscience and Mental Health, and Monash University). All volunteers were screened for MRI safety and enrolled into the study if they had no contraindications to MRI, consented to deidentified MRI data being publicly available, and were provided monetary compensation for travel and participation. Participants were included in the analysis if all MRI scans were acquired within a 6-month period. All scans were completed between September to December 2024, with an average interval of 17.8 days between the first and last scan (range: 2 to 64 days). Informed written consent was obtained from all participants for being included in the study, and ethical approval was granted by the University of Melbourne’s Human Research Ethics Committee (HREC #28396).

### 2.2 MRI scanner characteristics

Brain images were acquired on four 3T MRI scanners at three different sites across Melbourne, Australia (see Table 1). These included a 3T Magnetom Skyra, Vida, and two Prisma Fits (Siemens Healthcare, Erlangen, Germany). All systems were equipped with a 32-channel head coil.

**Table 1.** Site and scanner information.

	Scanner 1	Scanner 2	Scanner 3	Scanner 4
Site	Florey	Florey	MBI	MCRI
Vendor	Siemens	Siemens	Siemens	Siemens
Model	Prisma Fit	Vida	Skyra	Prisma Fit
Maximal gradient amplitude	80 mT/m	60 mT/m	45 mT/m	80 mT/m
Software version	VE11C	XA50	VE11C	XA30
Head coil	32 ch	32 ch	32 ch	32 ch

Florey: The Florey Institute of Neuroscience and Mental Health, Melbourne, Australia; MBI: Monash Biomedical Imaging, Monash University, Melbourne, Australia; MCRI: Murdoch Children’s Research Institute, Melbourne, Australia.

### 2.3 MRI data acquisition

Each MRI session lasted 1 hour, with approximately 40–50 minutes of scan time at each site. Diffusion-weighted images and structural images were acquired at each site, with all sites acquiring one consistent harmonization protocol for DWI, along with additional site-specific protocols not used in the present study.

The main harmonization protocol was a multi-shell DWI acquired with a spin-echo EPI sequence (TE/TR = 113/3345 ms, voxel size = 2 mm^3^, matrix size: 116 × 116 × 76, multi-band factor 4, no in-plane parallel acceleration). The complete diffusion gradient table consisted of: *b* = 0 s/mm^2^ (10 volumes), *b* = 300 s/mm^2^, (13 directions), *b* = 1000 s/mm^2^ (24 directions), *b* = 2000 s/mm^2^ (42 directions), and *b* = 3000 s/mm^2^ (60 directions). The diffusion encoding scheme was optimally split, with half the volumes acquired in the anterior-posterior (AP) phase-encoding direction, and half the volumes acquired in the posterior-anterior (PA) direction (designed to achieve optimal sampling across the AP and PA directions using a similar implementation originally developed for the developing Human Connectome Project (dHCP); Tournier et al., 2020). Acquisition time for this protocol was ~9 minutes.

### 2.4 DWI data processing

Here, we discuss the processing steps for the common DWI harmonization protocol.

All DWI data were preprocessed according to the recommended MRtrix3 pipeline for multi-tissue fixel-based analysis. This included MP-PCA denoising (Veraart et al., 2016), Gibbs ringing removal (Kellner et al., 2016), EPI susceptibility and motion correction using FSL topup and eddy (Andersson & Sotiropoulos, 2016; Andersson et al., 2003) with eddy QC (Bastiani et al., 2019), bias field correction using ANTs N4ITK (Tustison et al., 2010), and then upsampling all data to a voxel resolution of 1.25 mm^3^. For susceptibility field estimation, only leading *b* = 0 volumes were extracted and included, as interleaved *b* = 0 volumes were observed to contain residual eddy current distortions from prior volumes. Brain masks were estimated from the preprocessed diffusion-weighted image using both FSL brain extraction tool (BET; S. M. Smith, 2002) and MRtrix3 mask estimation, and we took the union brain mask for initial processing to ensure all brain regions were included for subsequent modelling.

**Fig. 1.**
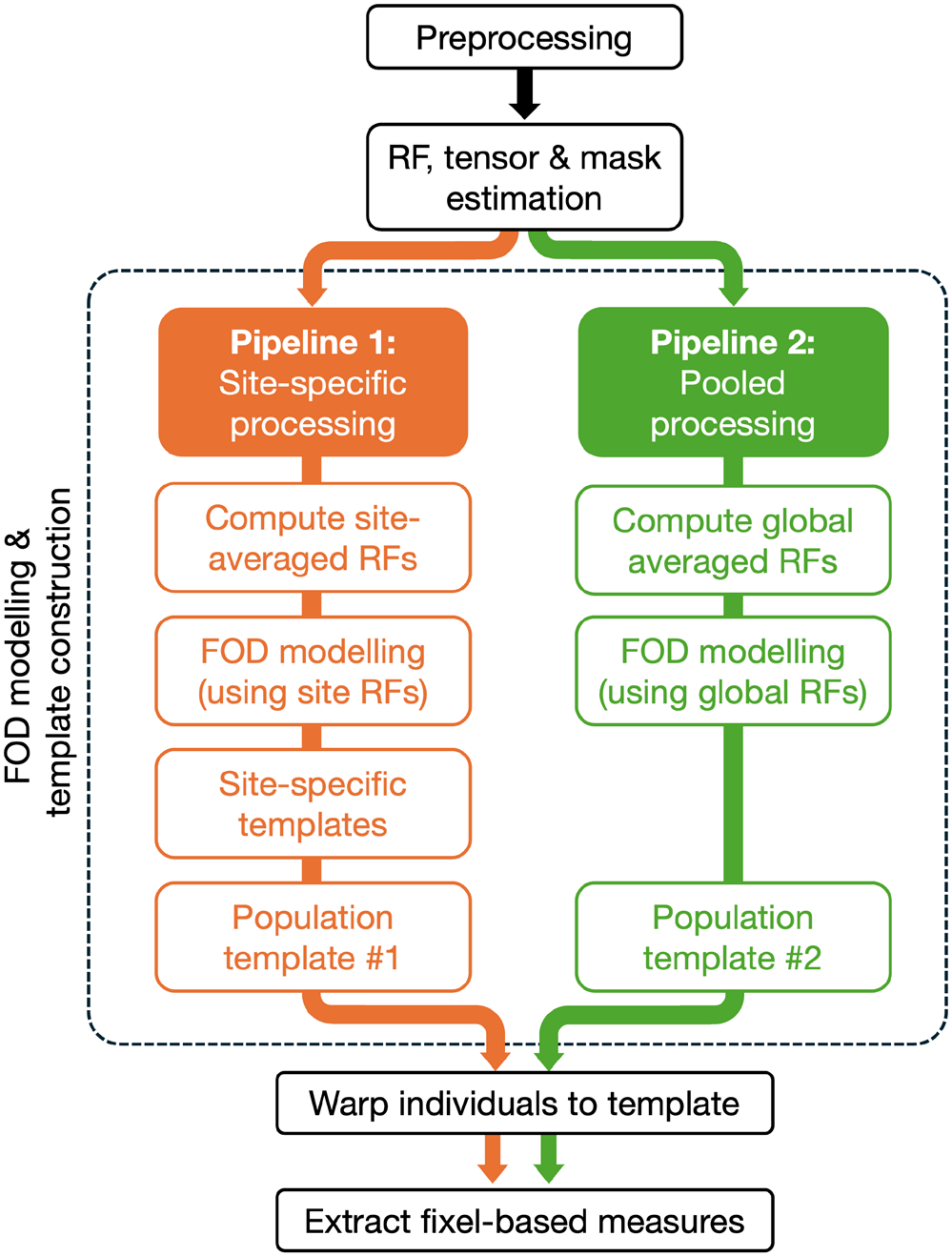
Processing pipelines for extracting fixel-based metrics. Here, we used two alternative approaches that might be taken when combining multi-site data. All data preprocessing, tensor fitting, brain mask estimation, and subject-specific response function estimation were performed in common (once per scanning session). Pipeline 1 describes the scenario where FOD modelling might be performed at a site-specific level (i.e., using site-averaged response functions (RFs) and site-specific templates). Pipeline 2 describes the scenario where data are pooled from different sites prior to FOD modelling (i.e., using globally averaged RFs and a global population template).

To mimic two potential strategies for data analysis when combining DWI data across sites for fixel-based analysis, we then processed the cohort using two distinct pipelines (see Fig. 1). The first pipeline (Pipeline 1: “site-specific processing”) processed DWI data for each site independently, only pooling data across sites for final analysis. The second pipeline (Pipeline 2: “pooled processing”) instead pooled DWI data across all sites for processing and analysis.

In both cases, after DWI data preprocessing, response functions were estimated for white matter, grey matter, and CSF (Dhollander et al., 2019) for each participant. Fibre orientation distribution (FOD) functions were then estimated using multi-shell multi-tissue CSD (MSMT-CSD) (Jeurissen et al., 2014). For the first pipeline, site-specific response functions (RFs) were computed (average response functions across all participants at a given site) and used for FOD estimation, while in the second pipeline, response functions averaged across all participants and scanning sessions (i.e., incorporating data from all individuals across all sites) were used for FOD modeling. For each pipeline, a study-specific FOD population template was built using an iterative registration and averaging approach (Raffelt et al., 2011), resulting in two distinct template spaces. For Pipeline 1, this was done by first building site-specific templates, then a common population template space using site-specific templates as input; for Pipeline 2, FOD images from all scanning sessions were used in a single template construction step. Each individual FOD image was then registered to the template using FOD-guided non-linear registration (Raffelt, Tournier, Crozier, et al., 2012).

Measures of fibre density (FD), fibre cross-section (FC), and fibre-density and cross-section (FDC) were then computed according to the fixel-based analysis pipeline (Raffelt et al., 2017). Whole-brain tractography was performed on the population template image using the iFOD2 tractography algorithm (Tournier et al., 2010) and default parameters for connectivity-based fixel enhancement (Raffelt et al., 2015), by first generating 20 million streamlines, which were subsequently filtered to 2 million streamlines by performing SIFT (R. E. Smith et al., 2013). Smoothing was applied to fixel-based measures using the fixel-fixel connectivity matrix computed from this tractogram (Raffelt et al., 2015).

While the primary purpose of this work was to examine fixel-based analysis metrics, we additionally assessed the reproducibility and reliability of more conventional tensor-based metrics: fractional anisotropy (FA) and mean diffusivity (MD). Here, the *b* = 1000 s/mm^2^ shell was extracted from the multi-shell harmonization dataset (to fit DTI modeling assumptions), and the tensor fit was performed using an iterated weighted least-squares approach (Veraart et al., 2013). FA and MD were estimated for each image, and then transformed to population template space (Population template #1; using non-linear transformations computed through registration of FOD images). To demonstrate reproducibility and reliability of FA and MD in the same template space as the fixel-based measures, voxel masks were computed as the set of all voxels containing at least one fixel, and tensor measures computed within those voxel masks. Given that these masks are more extensive than what is typically included within DTI-based analyses, we additionally computed reproducibility and reliability of these tensor measures within a DTI-specific white matter mask, including only template voxels with a mean FA > 0.2. For transparency, we additionally provide reproducibility and reliability findings for DTI measures derived from the full multi-shell data in the Supplementary Material. Of note, given that our data collection and processing were optimized for the purposes of fixel-based analysis, reproducibility and reliability of DTI findings cannot be directly compared to the FBA results, and we do not perform any statistical comparisons between these results.

We additionally investigated the reproducibility and reliability of fixel-based measures when computed from single-shell data. We extracted *b* = 1000, 2000, and 3000 s/mm^2^ shells from the full multi-shell dataset after preprocessing. Response functions were estimated for each *b*-shell, followed by FOD estimation using single-shell 3-tissue CSD (SS3T-CSD; using MRtrix3Tissue) (Dhollander & Connelly, 2016). As we used the same population template image for these analyses (Population template #1) and the fibre cross-section (FC) metric is a measure of deformation to template space, we examined only the reproducibility and reliability of the fibre density (FD) measure.

All processing steps (unless otherwise specified) were performed using MRtrix3 (version 3.0.4) (Tournier et al., 2019).

### 2.5 Tract segmentation

Tracts of interest were delineated by performing TractSeg (Wasserthal et al., 2018) on the population template FOD peaks image. Mean FD, FC, and FDC were computed for each tract, including only fixels with correspondence to the local tract direction. Mean voxel-based FA and MD were also computed for each tract, averaging across those voxels containing a fixel ascribed to the tract.

### 2.6 Statistical analysis

To assess the reproducibility and reliability of FBA and tensor-based metrics across the different sites, we computed the coefficients of variation (CV) and intraclass correlation coefficient (ICC).

CV is commonly used to assess the reproducibility of measures, and is broadly defined as the standard deviation of the group (σ) divided by the mean of the group (μ) (often expressed as a percentage). When assessing CV across sites with the same subjects, the within-subject variability (CV_ws_) is considered a more appropriate measure for reproducibility than pooled CV (Shoukri et al., 2008).

CV_ws_ was calculated following recommendations from the quantitative imaging biomarkers alliance (QIBA) for DWI biomarkers (Shukla-Dave et al., 2019). Here, squared CV_ws_ was computed for each subject (dividing variance by squared mean), and average squared CV_ws_ across subjects computed. CV_ws_ was, therefore, expressed as:

$$CV_{ws}=\sqrt{\frac{1}{n}\sum_{i=1}^{n}\left(\frac{\sigma_{i}^{2}}{\mu_{i}^{2}}\right)\times 100}$$

While we focus here on within-subject variability to assess reproducibility, we additionally computed between-subject CV (CV_bs_), which captures biological variability between subjects. CV_bs_ was computed by first taking the within-subject means (*µ*_*i*_), then computing the mean and standard deviations of these means across subjects:

$$CV_{bs}=\frac{\sigma_{\mu }}{\bar{\mu }}\times 100$$

Intraclass correlation coefficients (ICC) and their 95% confidence intervals were computed as two-way mixed effects, absolute agreement, single rater/measurement with formula ICC (A,1) (McGraw & Wong, 1996), calculated as:

$$ICC\left(A,1\right)=\;\frac{MS_{R}-\;MS_{E}}{MS_{R}+\left(k-1\right)MS_{E}+\;\frac{k}{n}\left(MS_{C}-MS_{E}\right)}$$

MS_R_ is the mean square for rows (i.e., subjects), MS_C_ is the mean square for columns (i.e., sites), and MS_E_ is the mean square for error. The choice of model (Model 3, two-way mixed) reflects assessment of specific sites included in this specific study, while the choice of form (single measurement) reflects the single measurement from each participant (Koo & Li, 2016; Trevethan, 2017). Finally, the ICC type is one that assesses *absolute agreement* between measurements (rather than *consistency*). We use the designation ICC (A,1) in line with McGraw and Wong (1996), noting that the same formulation is used for both random and mixed effects models assessing absolute agreement. This form is commonly reported as ICC (2,1) in many statistical packages.

While CV_ws_ captures the *reproducibility* of metrics, ICC was used to assess *reliability* of these measures (the overall consistency of measures across individuals) (Luque Laguna et al., 2020; Shoukri et al., 2008). Lower CV_ws_ values reflect better reproducibility, while higher ICC values reflect higher reliability. Generally, based on the 95% confidence interval, ICC values less than 0.5 indicate poor reliability, values between 0.5 and 0.75 reflect moderate reliability, values between 0.75 and 0.9 indicate good reliability, and values greater than 0.9 reflect excellent reliability (Koo & Li, 2016). However, we note that the interpretation of ICC depends upon the number of subjects and raters, and should be considered heuristic, rather than absolute.

CV_ws_ and ICC were assessed for each of the fixel-based measures (FD, FC, and FDC) at: (i) the whole brain level, where measures were averaged across all white matter fixels; (ii) at the tract level, where measures were averaged across tracts of interest; and (iii) at the fixel level, where reproducibility and reliability were assessed at each fixel in template space. These metrics were also computed for tensor-based scalars (FA and MD) at the same three levels.

Finally, to demonstrate the potential impact of measurement reliability, we provide sample size estimates in the case of an independent samples t-test for tract-level analyses (along with paired t-tests in the Supplementary Material). Here, to compute estimates of sample size requirements, we performed power calculations (at α = 0.05, power = 0.8) at different expected observable effect sizes (Cohen’s *d*), using a correction formula implemented in adjusting for group-level correlations (Baugh, 2002; Borneman, 2022), where:

$$d_{obs}=\;d_{true}\times \sqrt{ICC}$$

Here, ICC was used as a proxy for reliability, and the computed ICC values from the tract-level results were used. Three specific tracts (corticospinal tract (CST), arcuate fasciculus (AF), and fornix (FX)) were selected *a priori* for sample size calculation to capture different types of tracts. This was based on inclusion in previous repeatability work (Koller et al., 2021). This selection includes commonly studied tracts due to their clinical relevance (CST and AF), along with a tract that is more technically challenging to reconstruct consistently (FX).

## 3 Results

### 3.1 Pipeline characteristics

To assess reproducibility and reliability of fixel-based measures, we included two pipelines that might be adopted when performing fixel-based analyses across sites or scanners. Intermediary outputs from these two pipelines showed no obvious differences, with visual inspection of response functions, FOD images, and FOD templates exhibiting very high similarity. These intermediary outputs are provided in the Supplementary Material, including comparison of the derived response functions (Supplementary Fig. S1) and comparison between the two template spaces (Supplementary Fig. S2; Supplementary Table S1).

### 3.2 Reproducibility and reliability of fixel-based measures

#### 3.2.1 Comparing whole-brain averaged measures

Table 2 shows the reproducibility (indexed by CV_ws_) and reliability (indexed by ICC) of fixel-based measures (as well as DTI-based measures; see Section 3.3) when averaged across the whole-brain white matter. Across both pipelines, whole-brain averaged fixel-based measures demonstrated high reproducibility. Variability within subject (CV_ws_) was lower than between subject (CV_bs_) for all measures, ranging between 0.51% and 1.57% for within-subject, and 2.26% to 8.27% for between-subject variability. Whole-brain averaged fixel-based measures also demonstrated significant evidence of reliability (ICC p-value < 0.0001), with the FC and FDC metrics showing good to excellent reliability (ICC (A,1) > 0.9; ICC 95% confidence interval (CI) between 0.84 and 1.0 across both pipelines), while reliability for FD was slightly lower (ICC (A,1) > 0.75; ICC 95% CI between 0.34 and 0.95 across both pipelines). Reproducibility and reliability metrics were comparable between the two pipelines, though reliability tended to be slightly higher using the pooled processing approach (Table 2).

**Table 2.** Reproducibility and reliability of fixel-based and DTI-based measures across the whole-brain white matter.

METHOD	Metric	Mean	SD	CV_ws_ (%)	CV_bs_ (%)	ICC (95% CI)	ICC p-val
FBA (Pipeline 1)	FD	0.314	0.0075	1.18	2.26	0.782 (0.34, 0.94)	<0.0001
	FC	1.05	0.065	0.58	6.42	0.992 (0.97, 1.0)	<0.0001
	FDC	0.328	0.026	1.57	8.24	0.964 (0.84, 0.99)	<0.0001
FBA (Pipeline 2)	FD	0.313	0.0076	1.10	2.31	0.813 (0.42, 0.95)	<0.0001
	FC	1.05	0.065	0.51	6.40	0.994 (0.98, 1.0)	<0.0001
	FDC	0.330	0.027	1.41	8.27	0.971 (0.89, 0.99)	<0.0001
DTI (WM voxel mask)	FA	0.302	0.0068	1.24	2.07	0.733 (0.26, 0.93)	<0.0001
	MD	8.4e-04	1.5e-05	1.01	1.62	0.710 (0.32, 0.91)	<0.0001
DTI (FA mask)	FA	0.379	0.0094	1.50	2.19	0.679 (0.19, 0.91)	<0.0001
	MD	7.7e-04	1.2e-05	1.22	1.16	0.454 (0.08, 0.8)	<0.0001

Fixel-based analysis (FBA) metrics: FD: fibre density; FC: fibre bundle cross-section; FDC: fibre density and cross-section; all expressed in arbitrary units. Diffusion tensor imaging (DTI) based metrics: FA: fractional anisotropy (scalar ranging between 0 and 1); MD: mean diffusivity (expressed in mm^2^/s). Tensor-based metrics were averaged either using the voxel-based WM masks corresponding to the fixel mask (WM voxel mask) or using a DTI-specific voxel mask (template voxels with mean FA > 0.2). Mean and standard deviation (SD), within-subject coefficient of variation (CV_ws_), between-subject coefficient of variation (CV_bs_), and intraclass correlation coefficient (ICC) are reported for each metric. ICC values are reported using model 3 (two-way mixed effects), single measurement, absolute agreement (ICC (A,1)), alongside the ICC 95% confidence interval (CI) and *p*-value.

**Fig. 2.**
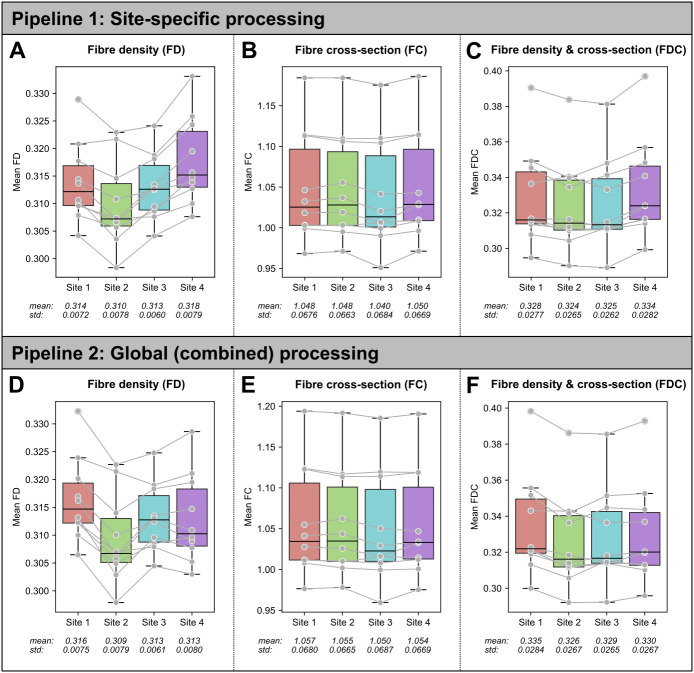
Reproducibility of mean fixel-based measures across the whole-brain. Boxplots show whole-brain fixel-wise measures: (A and D) fibre density (FD); (B and E) fibre cross-section (FC); (C and F) fibre density and cross-section (FDC). The top row shows results for Pipeline 1 (site-specific processing), and the bottom row shows results for Pipeline 2 (pooled processing). Points (in grey) represent whole-brain fixel-wise averages for each participant, with grey lines connecting points from the same individual across the four sites. Site-specific mean values and standard deviations (std) are shown for each measure.

Figure 2 shows boxplots of the mean fixel-based measures across the four sites.

#### 3.2.2 Comparing tract-level measures

When comparing tract-averaged fixel-based measures, reproducibility and reliability remained high across tracts. Reproducibility was high for the FD measure for both Pipeline 1 (CV_ws_ ranged between 0.74–3.63%) and Pipeline 2 (CV_ws_ ranged between 0.78–4.01%). Similarly, reproducibility was high for the FC metric (Pipeline 1 CV_ws_: 0.54–1.87%; Pipeline 2 CV_ws_: 0.60–1.76%) and for the FDC metric (Pipeline 1 CV_ws_: 1.23–5.03%; Pipeline 2 CV_ws_: 1.22–5.66%).

Of the fixel-based measures, reliability was most variable for the FD measure (Pipeline 1: ICC ranged between 0.538–0.963; Pipeline 2: ICC ranged between 0.488–0.973). Reliability was high across both pipelines for the FC measure (Pipeline 1 ICC: 0.939–0.996; Pipeline 2 ICC: 0.949–0.997), and the FDC measure (Pipeline 1 ICC: 0.813–0.989; Pipeline 2 ICC: 0.829–0.989).

**Fig. 3.**
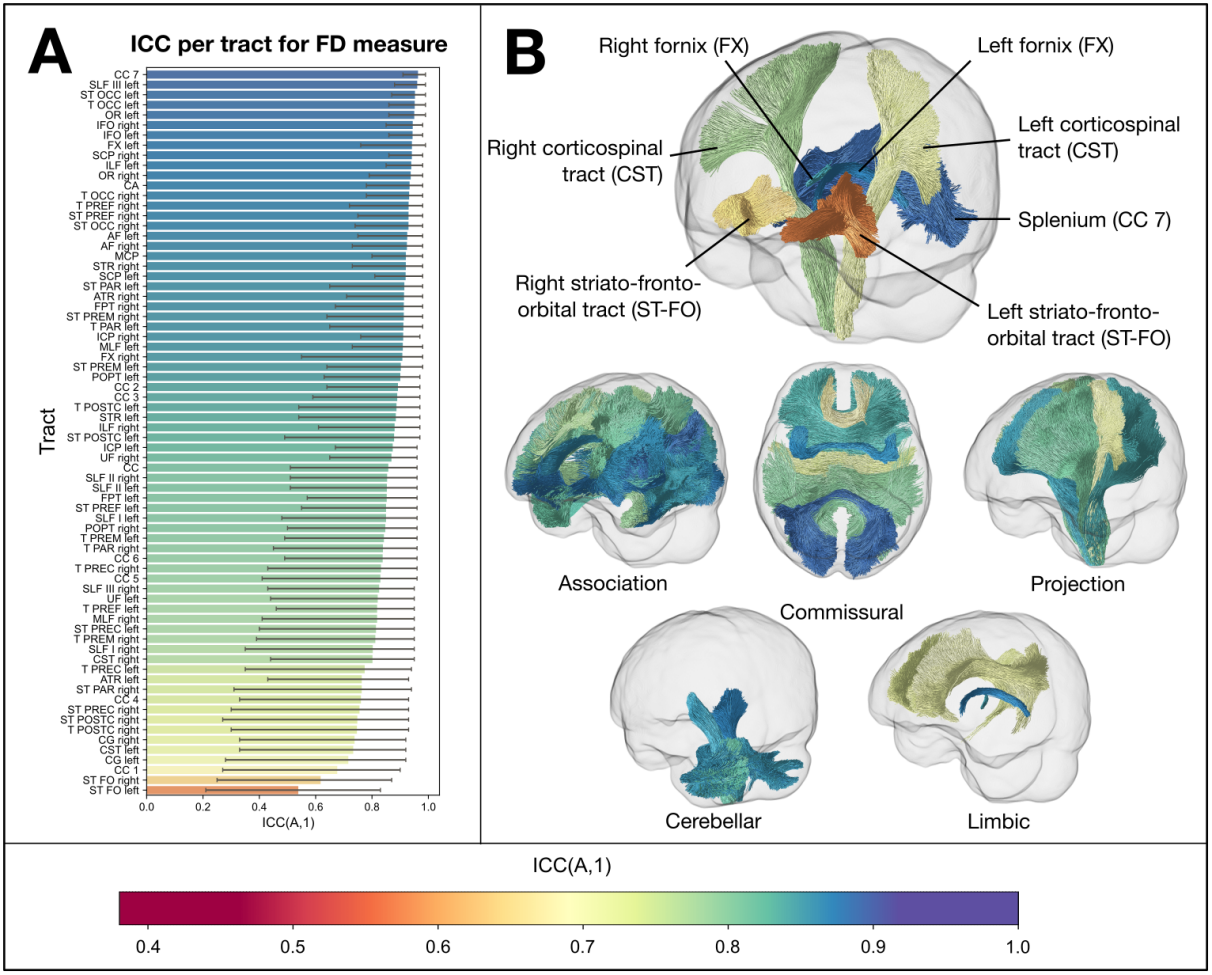
Intraclass correlation coefficient (ICC (A,1)) for the FD measure per tract using Pipeline 1. (A) Bar plot showing ICC for each tract (ordered from highest to lowest). Most tracts showed a very high ICC (>0.9), with the bilateral fornices (FX) showing the highest of all tracts, and bilateral striato-fronto-orbital tracts (STFO) showing the lowest ICC values. Error bars show 95% confidence interval of ICC. (B) Tract trajectories coloured by their ICC. The top image shows a number of key tracts, showing high (FX), moderate (corticospinal tracts; CST), and lower ICC (striato-fronto-orbital; ST-FO and rostrum of corpus callosum; CC 1). The bottom images show different ICC values for different tract classifications. See Wasserthal et al. (2018) for full tract definitions.

Figure 3 shows the ICC values for the FD measure for each white matter tract with Pipeline 1. We focus here on the FD metric, as it was the most affected by site of the fixel-based metrics. Of the 72 tracts included for analysis, 31 had an ICC > 0.9 (with 18 tracts showing good to excellent reliability based on the 95% CI); 33 had an ICC between 0.75 and 0.9, and the remaining 8 tracts an ICC between 0.5 and 0.75. Supplementary Figure S3 shows the ICC per tract for the FD measure with Pipeline 2 (same as Fig. 3, but with Pipeline 2).

Supplementary Table S2 summarizes tract-level reproducibility and reliability results, while Supplementary Tables S3–S5 report the reproducibility and reliability of fixel-based measures for each white matter tract with Pipeline 1 (site-specific processing).

#### 3.2.3 Comparing fixel-level measures

**Fig. 4.**
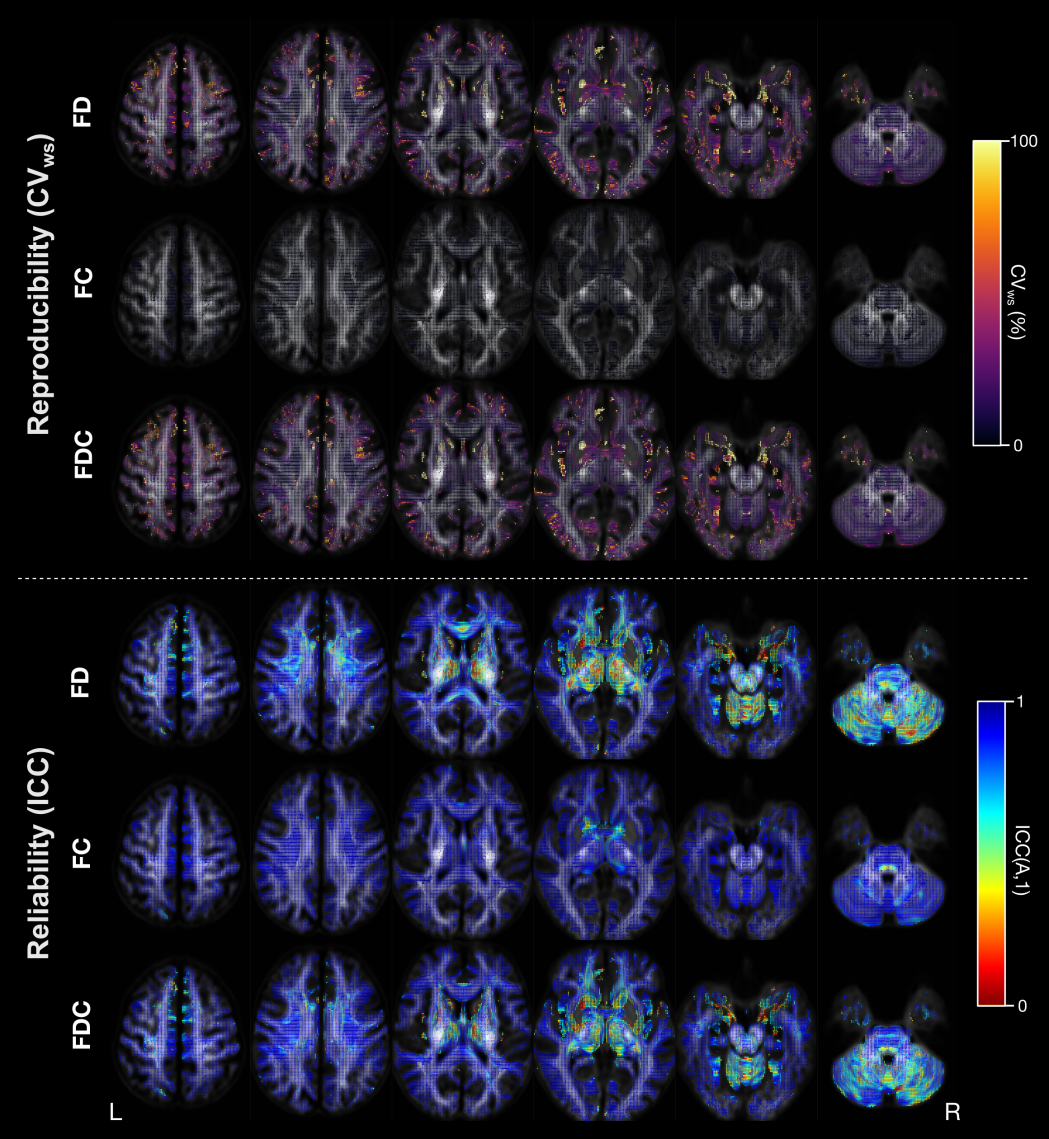
Reproducibility and reliability of FBA metrics at the fixel-level (Pipeline 1). Images show axial slices at 15 mm increments, with white matter fixels overlaid on the FOD template. Top panel: fixels are colored by their within-subject coefficient of variation (CVws) value, for the fibre density (FD; top), fibre cross-section (FC), and fibre density and cross-section (FDC) metrics. Bottom panel: fixels are colored by their ICC value, for FD (top), FC (middle), and FDC (bottom).

**Fig. 5.**
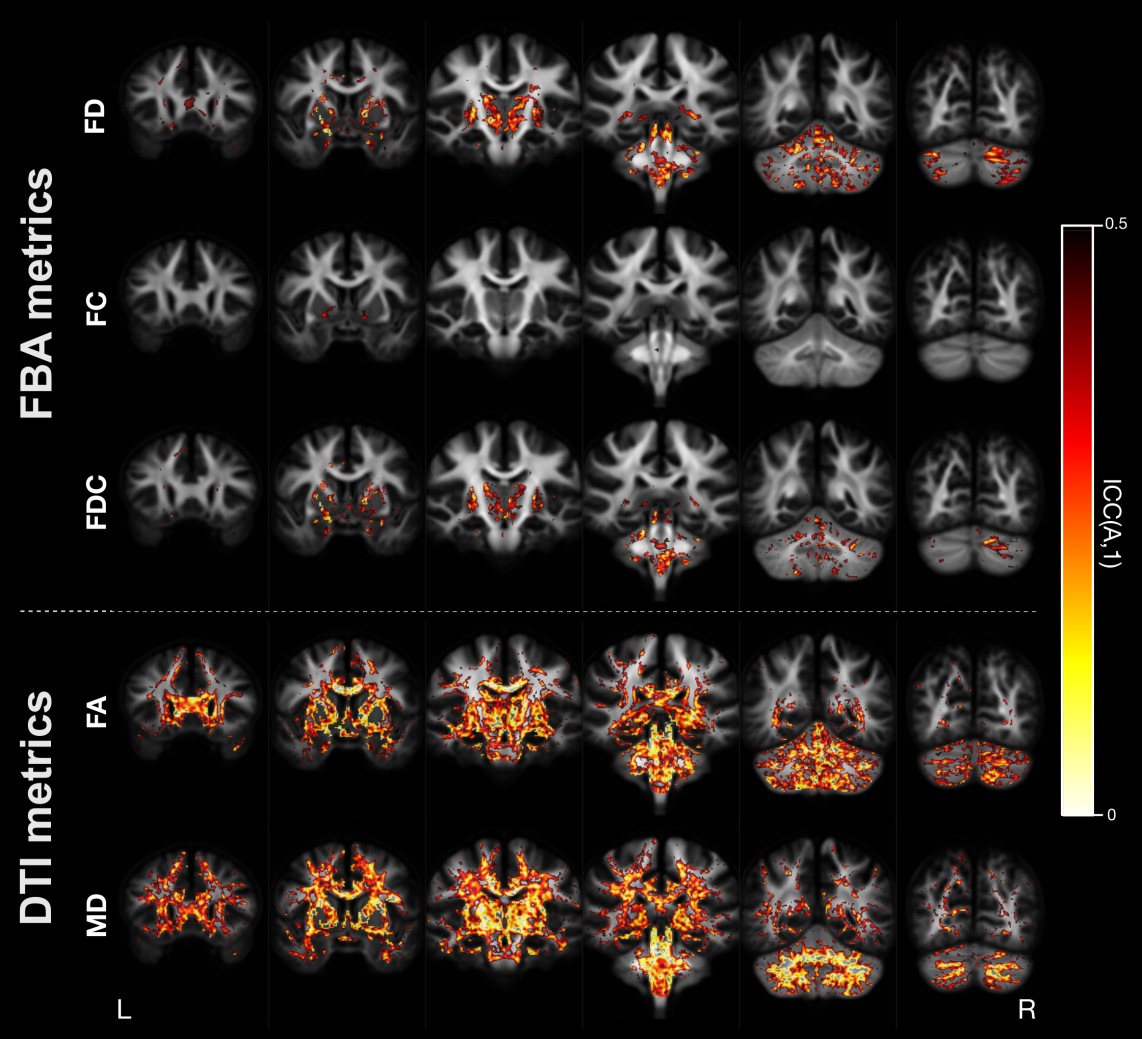
Thresholded ICC image showing regions of lower reliability. ICC is shown for fixel-based analysis (FBA) metrics (top) and tensor-based metrics (bottom). In both cases, the ICC images are thresholded to only show voxels with poor reliability (ICC < 0.5), with bright areas corresponding to lower ICC values and displayed on coronal slices at 18 mm increments. For FBA metrics, each voxel is colored by the minimum value of ICC across the fixels within that voxel (to enable easier visual comparison with tensor-based metrics).

Figure 4 shows reproducibility (CV_ws_) and reliability (ICC) for each individual fixel in template space (Pipeline 1), for each of the fixel-based measures. In general, reliability was high, with mean ICC across all fixels being 0.81 for FD, 0.95 for FC, and 0.84 for FDC (Table 3). Figure 5 shows a thresholded image to highlight regions with poor reliability for both FBA and tensor-based metrics (for which results are reported in 3.3). Fixels with lower reliability (ICC < 0.5) tended to be in cerebellar and subcortical grey matter regions, rather than in core white matter tracts. ICC was similar at the fixel-level when computing FODs using globally averaged response functions (Pipeline 2), with comparable patterns of spatial distribution of lower reliability regions to Pipeline 1 (see Table 3 for summary statistics on ICC across different pipelines and metrics, and Supplementary Figure S4 for ICC at each fixel for Pipeline 2).

**Table 3.** Summary statistics for ICC at the fixel- or voxel-level.

			ICC				
METHOD	Metric	Count	Mean	Median	SD	Min	Max
FBA (pipeline 1)	FD	627896	0.810	0.887	0.196	-0.326	0.99996
	FC	627896	0.946	0.964	0.056	0.0796	0.99762
	FDC	627896	0.844	0.909	0.169	-0.316	0.99943
FBA (pipeline 2)	FD	625296	0.810	0.888	0.198	-0.276	0.99962
	FC	625296	0.948	0.966	0.056	0.110	0.99810
	FDC	625296	0.844	0.911	0.170	-0.275	0.99963
DTI (WM voxel mask)	FA	431463	0.555	0.598	0.261	-0.3139	0.99392
	MD	431463	0.514	0.540	0.263	-0.3360	0.99056
DTI (FA mask)	FA	284920	0.549	0.589	0.262	-0.3019	0.99336
	MD	284920	0.447	0.453	0.255	-0.3360	0.99056

Table 4 shows the reproducibility (within-subject variability; CV_ws_) of fixel-based measures, summarized across all fixels within the white matter mask (density distribution plots shown in Supplementary Fig. S5). Overall, mean CV_ws_ was lowest for the FC measure (mean CV_ws_ < 3%), while substantially higher for the FD and FDC measures. Higher average CV_ws_ across all fixels for these two measures were likely driven by very large variability in a small number of fixels, with the mean CV_ws_ around 15%, and median CV_ws_ around 10% across all fixels. Figure 4 shows CV_ws_ for each of the metrics on axial slices. Core white matter pathways tended to have high reproducibility, while lower reproducibility (higher CV_ws_) was observed in the FD and FDC metrics in fixels closer to the cortical surface, and fixels crossing major white matter regions into subcortical regions (e.g., crossing fibre structures in the anterior limb of the internal capsule).

**Table 4.** Summary statistics for CV_ws_ at the fixel- or voxel-level.

			CV_ws_				
METHOD	Metric	Count	Mean	Median	SD	Min	Max
FBA (Pipeline 1)	FD	627896	15.95	10.82	18.87	0.59	200
	FC	627896	2.27	2.11	0.79	0.74	13.11
	FDC	627896	16.37	11.25	18.73	1.47	200
FBA (Pipeline 2)	FD	625296	16.05	10.79	19.24	0.82	200
	FC	625296	2.22	2.05	0.76	0.68	7.39
	FDC	625296	16.45	11.22	19.11	1.53	200
DTI (WM voxel mask)	FA	431463	20.43	20.62	8.84	1.75	64.37
	MD	431463	7.04	5.85	21.93	1.43	14101
DTI (FA mask)	FA	284920	17.42	16.71	8.20	1.75	64.37
	MD	284920	5.99	4.82	3.80	1.43	321.9

### 3.3 Reproducibility and reliability of tensor-based measures

In addition to comparing fixel-based measures, we also examined the reliability and reproducibility of tensor-based fractional anisotropy (FA) and mean diffusivity (MD). When using a white matter voxel mask equivalent to the fixel mask used for FBA analyses, whole-brain mean FA and MD showed high reproducibility, with low CV_ws_ (FA CV_ws_ = 1.24%, MD CV_ws_ = 1.01%). Reliability of mean FA and MD was comparable to that of the fixel-based FD measure, with ICC (A,1) of 0.73 and 0.71 respectively, with similarly large ranges in ICC 95% CI. Reliability and reproducibility were slightly lower when FA and MD were averaged across a constrained WM mask (see Table 2). Boxplots in Supplementary Figure S6 show whole-brain mean FA and MD for participants across sites.

**Fig. 6.**
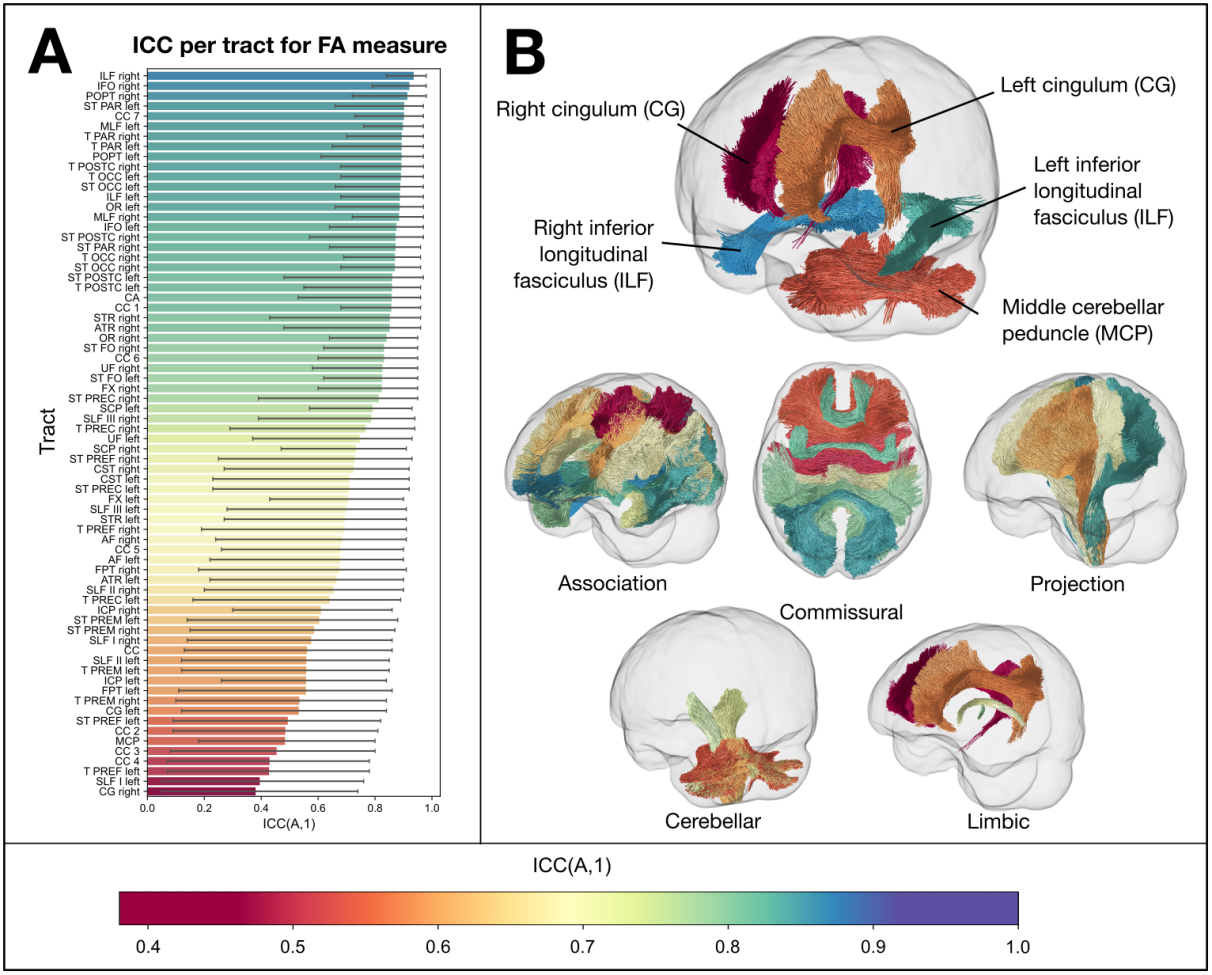
Intraclass correlation coefficient (ICC (A,1)) for the FA measure per tract. (A) Bar plot showing ICC for each tract (ordered from highest to lowest), using same colour scaling as Figure 3. Error bars show 95% confidence interval of ICC. (B) Glass brain images show tracts coloured by their ICC value, for a selection of tracts with the highest and lowest ICC (top), and for different tract classifications (bottom images). Here, reproducibility was lowest for the cerebellar white matter tracts (middle cerebellar peduncle (MCP) and bilateral cerebellar peduncles (ICP)), and highest in the motor system tracts and thalamic radiations.

Taking tract-averaged FA and MD using the same TractSeg tract definitions used for FBA metrics, we examined the reproducibility of measures averaged at each white matter tract. Within-subject variability (CV_ws_) for FA ranged from 0.65% to 4.58%, and intraclass correlation coefficient (ICC) from 0.380 to 0.936. The CV_ws_ for MD ranged from 0.90% to 1.98%, and ICC between 0.188 and 0.970. Figure 6 shows ICC for each of the TractSeg tracts for the FA measure. In general, the pattern of tracts showing lower/higher reliability for FA did not necessarily overlap with the FD measure (see Fig. 3 for comparison). Supplementary Tables S6 and S7 show results for each tract for FA and MD, respectively.

When examining ICC across white matter voxels independently, a similar pattern was observed to fixel-based measures, with ICC lowest in cerebellar and subcortical white matter regions. However, mean ICC was substantially lower for tensor-based metrics than for fixel-based measures, with mean ICC across all WM voxels at 0.56 for FA and 0.51 for MD (Table 3). Given that the fixel-based white matter mask may be more extensive than white matter masks used for DTI-based analyses, we also computed a DTI-specific white matter mask (constraining to voxels where the FA > 0.2). However, results were similar when using the constrained WM mask, with mean ICC across all WM voxels at 0.55 for FA, and 0.45 for MD. Figure 5 shows voxels with poor reliability for both the fixel-based and tensor-based measures, demonstrating the greater extent of lower reliability voxels in tensor-based metrics, which included some key white matter regions (e.g., corpus callosum, internal capsule). Some voxels within these same regions exhibited negative ICC values, likely due to low between-subject variance.

The reproducibility of tensor-based metrics at the voxel level was generally high within core white matter tracts, with CV_ws_ similar to that of FBA metrics. Supplementary Figure S5 shows distribution of voxel-wise ICC and CV_ws_ for all metrics, while Supplementary Figure S6 shows ICC and CV_ws_ for the FA and MD measures on a single axial slice, alongside whole-brain boxplots. Supplementary Figure S7 additionally shows CV_ws_ and ICC of FA and MD across the whole brain for transparency, and Supplementary Figure S8 shows the difference between the included brain masks.

Reproducibility and reliability of tensor-based metrics when derived from the full multi-shell dataset are also available in the Supplementary Material (Supplementary Tables S8–S11; Supplementary Fig. S9).

### 3.4 Reproducibility of fiber density measure at different b-values

The fibre density (FD) measure is known to be dependent on *b*-value, and as such, we also examined whether reproducibility of this measure would be different at different *b*-values (see Supplementary Fig. S10). Here, we extracted each *b*-shell from the full multi-shell DWI, and modeled FODs using the single-shell data only, using single-shell 3-tissue CSD (SS3T-CSD). The FD measure extracted with this approach was, indeed, dependent on *b*-value, with higher mean FD across the whole brain at *b* = 1000 s/mm^2^ and *b* = 2000 s/mm^2^ than at *b* = 3000 s/mm^2^. Reliability of the FD measure was high across all *b*-shells, though it was highest when using the *b* = 3000 s/mm^2^ data (ICC of 0.836; 95% CI: [0.54, 0.95]), followed by the *b* = 1000 s/mm^2^ data (ICC of 0.828; 95% CI: [0.5, 0.95]), and lowest for the *b* = 2000 s/mm^2^ shell (ICC of 0.787; 95% CI: [0.47, 0.94]) (see Supplementary Table S12). Reproducibility of FD was high across all *b*-shells, with all demonstrating within-subject variability (CV_ws_) around 1%. Reproducibility and reliability metrics at the tract-averaged and fixel level at different *b*-values are also summarized in the Supplementary Material (Supplementary Tables S13 and S14).

### 3.5 Sample size estimates for tract-level analyses

**Fig. 7.**
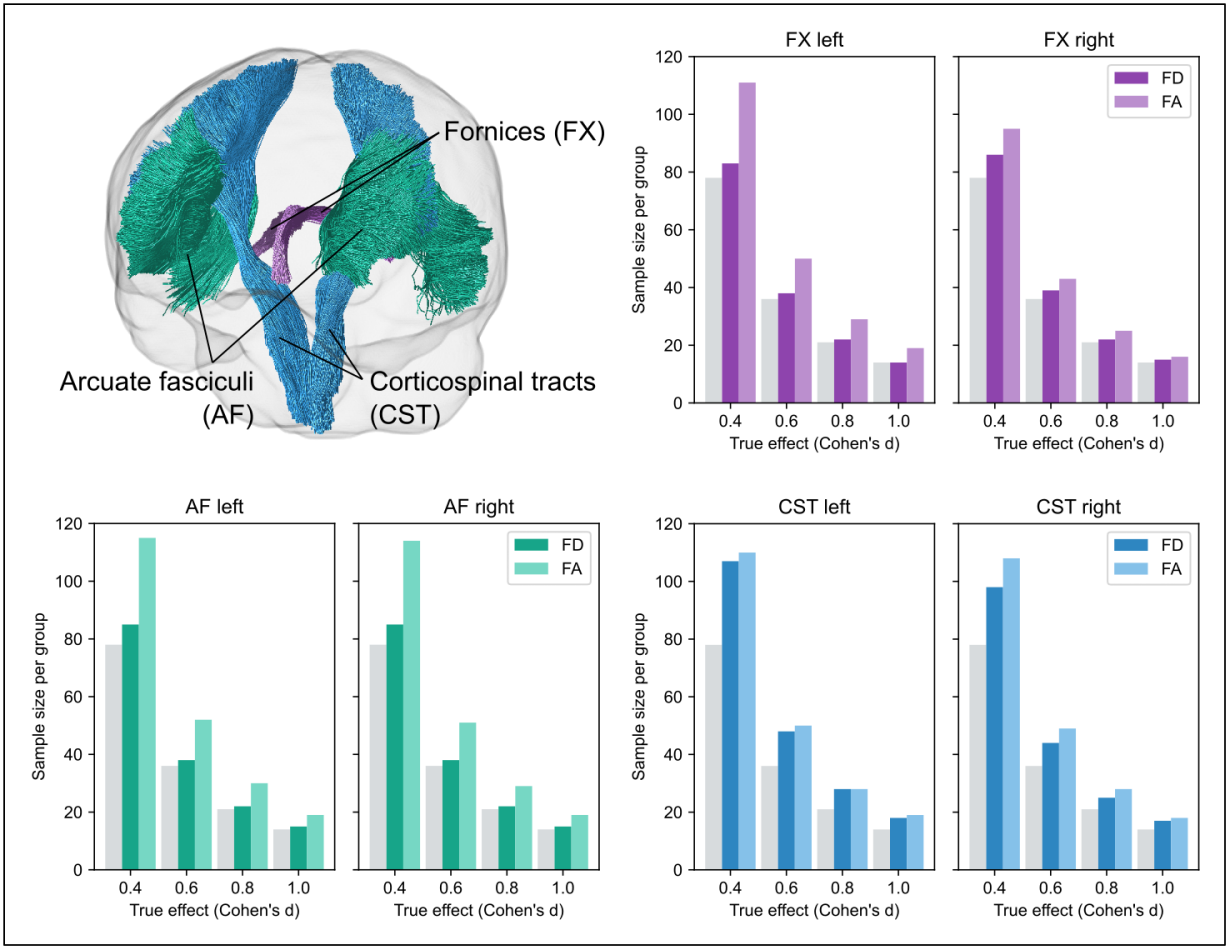
Sample size estimates for tract-level analyses for fibre density (FD) and fractional anisotropy (FA). Glass brain in top left shows the three tracts selected for sample size estimates (fornix (FX); arcuate fasciculus (AF); corticospinal tract (CST)). Bar plots show sample size requirements for each of these tracts, using the ICC values that were computed from the tract-level reproducibility analyses. Light grey bars show sample size requirements using true effect size (without adjustment for measurement reliability).

To demonstrate the potential impact of reliability on studies, sample size estimates were computed for tract-level analyses, by using the ICC values reported in Sections 3.2.2 and 3.3, estimating observable effect sizes at different expected true effects. Figure 7 shows sample size requirements per group for an independent sample t-test, with α = 0.05 and power = 0.8, for three select tracts (fornix, arcuate fasciculus, and corticospinal tract), for both the fibre density (FD) and fractional anisotropy (FA) metrics across a range of expected true effect sizes (0.4 < d < 1.0). Sample size requirements were similar for fixel-based FD to tensor-based FA, but smaller tracts like the fornix had lower sample size requirements for FD, due to the greater impact of site variability on the FA measure than for the FD measure.

Of note, these sample size estimates are based on *independent* tract analyses. More stringent α thresholds that might be used when conducting multiple tract analyses would result in higher sample size requirements. We additionally provide sample size estimates for paired t-tests in the Supplementary Material (Fig. S11).

## 4 Discussion

In this work, we present the TRAMFIX dataset, a traveling heads dataset designed to probe the reproducibility and reliability of fixel-based analysis (FBA) metrics derived from DWI data. We demonstrate that whole-brain and tract-averaged FBA measures are highly reproducible and reliable across scanners when using consistent protocols. However, when examining reproducibility and reliability at individual fixels, it was evident that certain white matter tracts and pathways are more greatly impacted by scanner variability than others, which may be underappreciated at the whole-brain and tract-averaged level. This highlights the importance of careful interpretation in certain brain regions when conducting multi-site studies, and the potential need for appropriate harmonization approaches. Of the three fixel-based metrics, fibre cross-section (FC) tended to be the most robust to scanner differences, while fibre density (FD) tended to exhibit lower reliability, and the combined fibre density and cross-section measure (FDC) tended to exhibit the lower reproducibility. Of note, both reproducibility and reliability of FBA metrics was comparable, if not higher, than DTI-based measures derived from a subset of the same data, evident particularly in subcortical and cerebellar white matter, as well as in specific white matter tracts. However, we also highlight that the *b*-value dependence of fixel-based measures may pose an additional challenge when pooling data across different protocols. We additionally demonstrate the potential impact of metric reliability on sample size estimates. Together, our findings provide novel insight into the reproducibility and reliability of fixel-based measures, which will potentially guide future studies that expand FBA beyond the single-site, and into multi-site designs.

### 4.1 Reproducibility and reliability of FBA measures

There has been growing interest in the repeatability, reproducibility, and reliability of DWI-derived measures, as quantitative MRI measures are known to demonstrate some level of variability even under identical conditions (Jansen et al., 2007; Koller et al., 2021; Vollmar et al., 2010), and to be highly susceptible to differences in scanner hardware and data acquisition (Mirzaalian et al., 2016; Tax et al., 2019). There have now been several studies examining the reproducibility and reliability of tensor-based metrics (Besseling et al., 2012; Boukadi et al., 2019; Koller et al., 2021; Luque Laguna et al., 2020; Magnotta et al., 2012; Palacios et al., 2017; Vollmar et al., 2010), measures from multicompartment models (Andica et al., 2020; Fan et al., 2021; Veraart et al., 2021), graph theoretical measures (Roine et al., 2019), and fiber tractography (Boukadi et al., 2019; Schilling et al., 2021). This work extends our understanding of DWI reproducibility and reliability to fixel-based metrics, which are being increasingly adopted in research studies due to their ability to identify fiber tract-specific differences in clinical cohorts. Our findings demonstrate that FBA measures are highly reproducible and reliable across different scanners and sites when using an identical imaging protocol. FBA metrics demonstrated lower within-subject variation (CV_ws_) than between-subject variation (CV_bs_), and high intraclass correlation coefficient (ICC) across the different scanners, with comparable results to previous literature in other DWI metrics. Within our study, we similarly observed comparable reproducibility in FBA metrics as with DTI-based FA and MD, with slightly higher reliability evident for FBA measures particularly at the tract- and voxel-level.

Our findings may be taken to support the broader application of fixel-based analysis studies beyond predominantly single-site into multi-site study designs. Indeed, reliability and reproducibility of fixel-based measures was high when looking at whole-brain averaged or tract-averaged metrics, with high ICC values and low within-subject variability across most white matter bundles. This was in keeping with other DWI metrics, where inter-scanner variabilities have generally been reported to be less than 10% within select regions or tracts of interest (Cai et al., 2021; Grech-Sollars et al., 2015; Palacios et al., 2017; Vollmar et al., 2010). DTI-based studies have increasingly been expanded to multi-site designs, and the comparable reproducibility of FBA metrics reported here demonstrates promise for studies that may wish to explore fiber-specific white matter differences in cohorts with multi-site data collection. To this end, we provide sample size estimates in the context of examining tract-specific differences in multi-site cohorts, to highlight the potential impact of measurement reliability in multi-site designs. However, we do recommend caution when interpreting findings in brain regions that may be more susceptible to scanner differences.

Importantly, we additionally examined reliability and reproducibility of fixel-based metrics at the individual fixel-level (as well as DTI metrics at the voxel-level) in this study. Of note, previous studies examining variability of DWI measures across sessions or sites have predominantly focused on quantifying reliability (ICC) or reproducibility (CV_ws_) in whole-brain, tract-, or ROI-averaged metrics. While this is a valuable way to assess and summarise DWI metrics, many clinical research studies aim to conduct group analyses at the more granular voxel-level (or in the case of FBA, at the fixel-level). Studies that have examined variability in DWI metrics at the voxel-level have generally assessed correlation (using Pearson’s R) or absolute deviation between paired tests (e.g., test-retest pairs) (Fan et al., 2021; Koller et al., 2021).

Here, we provide ICC and CV_ws_ estimates at each individual fixel or voxel, enabling visualization of the exact brain areas most affected by scanner differences. Indeed, our fixel- and voxel-level results indicated clear spatial patterns to reliability, with much lower ICC in certain brain areas than was observable at the tract or whole-brain averaged level. This included fixels and voxels with negative ICC values, likely arising due to higher within-subject variability than between-subject variability in these regions (Lahey et al., 1983), which could be more common in small sample cohorts (Liljequist et al., 2019). The spatial pattern of low ICC within subcortical structures replicates previous reports that have shown lower ICC in voxel-based measures (Luque Laguna et al., 2020). Areas of low reliability (Fig. 5) were predominantly in the subcortex (white matter fixels within grey matter structures, including thalamus, subthalamus, hypothalamus, lentiform nucleus) and posterior fossa (brainstem, cerebellar folia and cerebellar white matter), which could be the result of noisier FODs at the grey-white matter interface (De Luca et al., 2020), poorer FOD-based registration, or scanner-related differences in achievable SNR in these regions. EPI susceptibility distortion could also drive lower reproducibility and reliability in certain brain regions, including in the mesial temporal lobes, where proximity to ethmoid sinuses may have resulted in the observed lower reproducibility and reliability. However, we also note that for DTI-based measures, lower ICC was observed across many deep white matter voxels, in line with previous findings demonstrating lower reliability in DTI metrics in some key white matter regions on a single scanner (Luque Laguna et al., 2020; Vollmar et al., 2010). Reproducibility tended to be high across most white matter fixels for FBA metrics, and voxels for tensor-based metrics, with greater within-subject variability at cortical boundaries or in minor crossing fixels. This was similarly in keeping with studies that have reported high reproducibility (low CV) in core white matter at the voxel-level for tensor-based metrics (Luque Laguna et al., 2020; Vollmar et al., 2010). Providing estimates of reliability and reproducibility at the individual voxel- or fixel-level may be informative for studies conducting multi-site DWI analyses, as many research studies perform analyses at this granularity.

Of note, we investigated two potential pipelines that might be used when pooling data for fixel-based analyses across sites. Modeling data using constrained spherical deconvolution (CSD) relies on first defining a response function (generally estimated from the DWI data at hand). As fiber orientation distribution functions are then estimated from the derived response functions (by deconvolution of the response function from the measured DWI signal (Tournier et al., 2007), the use of standardized (for example, group-averaged) response functions is recommended when performing fixel-based analysis (Raffelt, Tournier, Rose, et al., 2012; Raffelt et al., 2017). We tested reproducibility and reliability of fixel-based measures derived from FODs estimated using response functions derived in one of two ways: either site-specific aggregation, or pooling across the entire study. Although we expected combined processing (FOD estimation using RFs pooled across the whole study) would reduce variability and improve reproducibility and reliability across sites, results were, in fact, highly similar across the two pipelines. This suggests that when combining data for FBA across multiple sites, data processing (that is, DWI modeling steps prior to template registration) could reasonably be performed at a site-specific level prior to pooling data across sites for further analysis (coregistration to a common template, and statistical analysis). However, we note that for this particular study, DWI data were collected using identical protocols with matched parameters across all sites, enabling cohort-wide pooled processing (which may not be possible in other contexts). The use of matched protocols, along with the inclusion of scanners from the same vendor, resulted in low variability across sites (CV_ws_ < 5% across all white matter tracts). Where there is greater variance across sites, group-based processing may still help to improve reproducibility and reliability of fixel-based measures. We also note that ANOVA-based ICC relies on independence of measurements (Kenny & Judd, 1986), and the use of pooled response functions may result in some interdependence across scans. Here, we note that ICC measures should be interpreted with some caution, alongside reproducibility findings.

Finally, while we observed fairly high reproducibility and reliability for fixel-based metrics, there was an observable effect of site on these measures, evident even at the whole-brain averaged level (Fig. 2). These were apparent even when using a pooled processing approach for FBA (Pipeline 2) that might be expected to reduce variability across sites, in the presence of an identical protocol, suggesting that these may reflect inherent scanner differences. Indeed, while we attempted to control for a number of factors likely to influence quantitative diffusion-based metrics (voxel size, *b*-values, data processing and modeling, etc.), scanner-specific factors (e.g., SNR and gradient linearity, scanner vibrations, image registration) may explain observed differences (Jones, 2010). By assessing reproducibility and reliability at different granularities, we were able to highlight certain regions that may drive observed variability; however, future work is needed to further assess potential causes of site-related variability.

### 4.2 Assessing reproducibility and reliability

To assess reproducibility and reliability, we made use of two key statistical metrics that have similarly been implemented in previous work. When assessing reproducibility of diffusion-based measures, coefficient of variation is often used (Cai et al., 2021; Grech-Sollars et al., 2015; Koller et al., 2021; Luque Laguna et al., 2020; Magnotta et al., 2012; Pfefferbaum et al., 2003; Vollmar et al., 2010). Although CV is quite simply defined as the standard deviation of measurements divided by the mean, when used to assess reproducibility across individuals and sites, separating the variability into within-subject and between-subject variability is valuable for differentiating site-related variability (i.e., reproducibility) from between-subject differences (Shoukri et al., 2008). However, even when computing CV_ws_, studies will often use a pooled (grand) mean (Kurokawa et al., 2021; Luque Laguna et al., 2020), which is still influenced by data across subjects. Here, we utilized a robust measure of CV_ws_, as recommended by the quantitative imaging biomarkers alliance (QIBA) (Shukla-Dave et al., 2019), and advocate for future studies to similarly quantify reproducibility in this way.

Reliability, on the other hand, is commonly assessed using the intraclass correlation coefficient (ICC). Here, we note that both choice of ICC model, form and type, along with appropriate interpretation of the ICC is important (see Trevethan (2017) and Koo & Li (2016) for helpful commentary on this topic). We report absolute agreement ICC type (ICC (A,1) by McGraw & Wong (1996) convention) to assess reliability based on the actual values of quantitative data, rather than consistent spread across individuals. Of note, the formula used to compute ICC (A,1) here is identical to ICC (2,1) by Shrout and Fleiss (1979) convention. Prior studies assessing reliability of DWI-derived metrics have used different forms of ICC, with some using formulations reflecting consistency types (Besseling et al., 2012; Luque Laguna et al., 2020; Roine et al., 2019; Vollmar et al., 2010), which are likely to result in higher ICC values than absolute agreement types reported by others (Boukadi et al., 2019; Koller et al., 2021). Of note, absolute agreement ICCs are more appropriate in contexts when assessing quantitative measures than consistency ICCs (Trevethan, 2017), particularly in test-retest scenarios (Koo & Li, 2016). Choice of ICC model and form should be also made based on study design (e.g., test-retest repeatability vs. traveling heads). In addition, we highlight recommendations to report and interpret ICC based on the 95% confidence interval when assessing reliability (Koo & Li, 2016). In addition, ANOVA-based confidence intervals based on larger sample normal approximations may not hold for small sample cohorts such as this, and the use of bootstrapped confidence intervals may be more appropriate. Finally, we echo previous recommendations to assess both *reproducibility* (generally indexed with within-subject variability) and *reliability* (indexed with ICC) in such studies, as they reflect separate properties (Luque Laguna et al., 2020; Shoukri et al., 2008).

### 4.3 Limitations and future directions

To our knowledge, this is the first study to probe the reproducibility and reliability of fixel-based analysis (FBA) measures, making use of a traveling heads cohort. Here, we were particularly interested in assessing scanner-related variability across local neuroimaging research centers, and, as such, focused on these inter-scanner differences while making use of an identical harmonized DWI protocol across sites (i.e., we focused on *reproducibility* rather than *repeatability*; Plesser, 2018; Raunig et al., 2015). We were unable to assess inter-session (test-retest) repeatability, as well as potential protocol-related variability in fixel-based measures. Variations in diffusion acquisition protocols, including the *b*-value and number of gradient directions, have been widely demonstrated to impact diffusion tensor-based measures (Chung et al., 2013; Jones, 2004; Papadakis et al., 1999; Skare et al., 2000; Tournier et al., 2011). As has been previously demonstrated (Genc, Tax, et al., 2020), we also note the *b*-value dependence of the apparent fibre density (AFD) measure, highlighting that protocol-related variability may be much more pronounced than scanner-related differences.

We also note that the use of multi-shell or single-shell data and subsequent FOD modelling could result in further variability in derived metrics (e.g., modeling data with multi-shell multi-tissue constrained spherical deconvolution (CSD) vs. single-shell single-tissue, or 3-tissue CSD)). In addition, while we focused on two pipelines that might be used when performing multi-site fixel-based analyses, making use of pooled response functions for FOD modeling (as recommended for quantitative analyses such as FBA), the use of individual response functions to estimate FODs may result in further variability. Further work examining reproducibility and reliability of fixel-based measures using different modeling approaches is likely to be insightful. Future work could also assess the impact of alternative approaches along the fixel-based analysis pipeline, including stricter constraints to fixel inclusion, or approaches that improve correspondence of fixels (and therefore derived quantitative metrics) across participants (Rafipoor et al., 2025; R. E. Smith & Connelly, 2018).

This work focused on assessing the reproducibility and reliability of fixel-based measures, rather than other DWI metrics. As such, our acquisition and analysis pipeline was designed specifically for the purposes of how researchers might typically perform fixel-based analyses. While we included DTI-based metrics for the purposes of comparison with previous DTI studies, we highlight that direct comparison of reproducibility and reliability between DTI and FBA metrics is challenging, given differences in acquisition requirements, modelling assumptions, and analysis granularity (voxel- vs. fixel-level). Indeed, data that might be ideal for one model would inherently not meet the modeling assumptions for the other, meaning that the same data cannot be fairly used to compare the two approaches. In this study, we primarily modeled tensor-based metrics using only a subset of the multi-shell data used to extract FBA measures (extracting the *b* = 1000 s/mm^2^ shell to fit modeling assumptions of the tensor (Armitage & Bastin, 2001; Jones, 2014; Kingsley & Monahan, 2004); although we additionally provide results derived when modeling DTI measures using the full multi-shell data in the Supplementary Material). Our acquisition was not optimized for the purposes of DTI and included a longer echo time than necessary for a typical DTI study (due to the collection of higher *b*-shells). As such, we cannot directly compare the reproducibility and reliability of DTI-based measures and fixel-based measures. Nonetheless, it is promising that our DTI-based findings are largely in line with previous studies examining reproducibility and reliability of tensor metrics (Koller et al., 2021; Vollmar et al., 2010), with similar ICC values when examining tract-averaged DTI measures to single-site designs (Boukadi et al., 2019), and similar spatial patterns to regions of higher and lower reproducibility and reliability (Luque Laguna et al., 2020).

Our work was also limited by the inclusion of scanners from a single vendor. Previous work has highlighted that vendor-related variability in diffusion-derived features may be much greater than that from different scanner models of the same vendor (Andica et al., 2020; Schilling et al., 2021). Of note, even with identical protocols and scanner model (Scanner 1 and 4), we observed some variability in derived measures, as has been previously reported for DTI-based metrics. Future work probing the impact of different vendors on fixel-based measures will be valuable.

Finally, to bring FBA studies into multi-site designs, future work evaluating the performance of different harmonization approaches may be valuable. Our analysis of repeatability and reproducibility excluded data harmonization approaches that are becoming increasingly prevalent in this domain. The TRAMFIX dataset contains fewer acquisitions than is conventionally considered adequate to perform such harmonization (Cetin Karayumak et al., 2019), precluding its inclusion in the present work; however, future work evaluating the performance of different harmonization approaches may nonetheless be valuable. There are now a range of potential diffusion harmonization approaches available (Pinto et al., 2020; Tax et al., 2019), including those that harmonize on the diffusion signal (Mirzaalian et al., 2016), and batch harmonization approaches for derived metrics (Fortin et al., 2017). These may be particularly pertinent when performing whole-brain analyses rather than examining tract-averaged metrics, given the variable reliability across fixels. The performance of batch harmonization approaches on fixel-based measures has been characterized to some extent (Mito et al., 2023; Zou et al., 2025), while other harmonization approaches have not yet been widely explored. Traveling participant datasets can be valuable in this way, as the intrinsic biological variability of the data is minimized when assessing performance of different harmonization approaches.

We hope that the TRAMFIX dataset will be a valuable resource to future studies that may wish to further probe the reproducibility and reliability of fixel-based measures and associated processing pipelines, as well as the performance of harmonization techniques. We provide this as a resource alongside other traveling participant and test-retest datasets available in the community (Cai et al., 2021; Koller et al., 2021; Kurokawa et al., 2021; Tax et al., 2019).

### 4.4 Conclusion

Here, we present traveling heads data for fixel-based metrics—the TRAMFIX dataset (TRavelling Across Melbourne for FIXel-based analysis). We demonstrate high reliability and reproducibility of fixel-based measures across scanners from the same vendor and sites, when using identical protocols, with similar results to that reported from other DWI measures. These findings may help to guide large multi-site study designs, and improve the reach of more advanced approaches such as Fixel-Based Analysis. Future studies that examine the impact of different protocols as well as performance of data harmonization approaches are likely to be valuable. To this end, we provide the TRAMFIX dataset as a public resource for future work.

## Supplementary Material

Supplementary Material

## Data Availability

The TRAMFIX dataset is available to the community as a resource. Data have been uploaded to OpenNeuro (https://openneuro.org/datasets/ds006935). The code used to process DWI data, and to perform statistical analyses is available on GitHub (https://github.com/remikamito/TRAMFIX).
