# Supplementary material for: TRAMFIX: TRavelling Across Melbourne for FIXel-based analysis (a reproducibility and reliability study)
Source: Imaging Neurosci (Camb). 2026 Mar 10;4:IMAG.a.1153. doi: 10.1162/IMAG.a.1153 (PMC12977090; doi:10.1162/IMAG.a.1153)
Supplement: Supplementary Material [file IMAG.a.1153_supp.pdf]

Supplementary Material

Comparison between pipelines and templates (Supplement to Section 3.1)

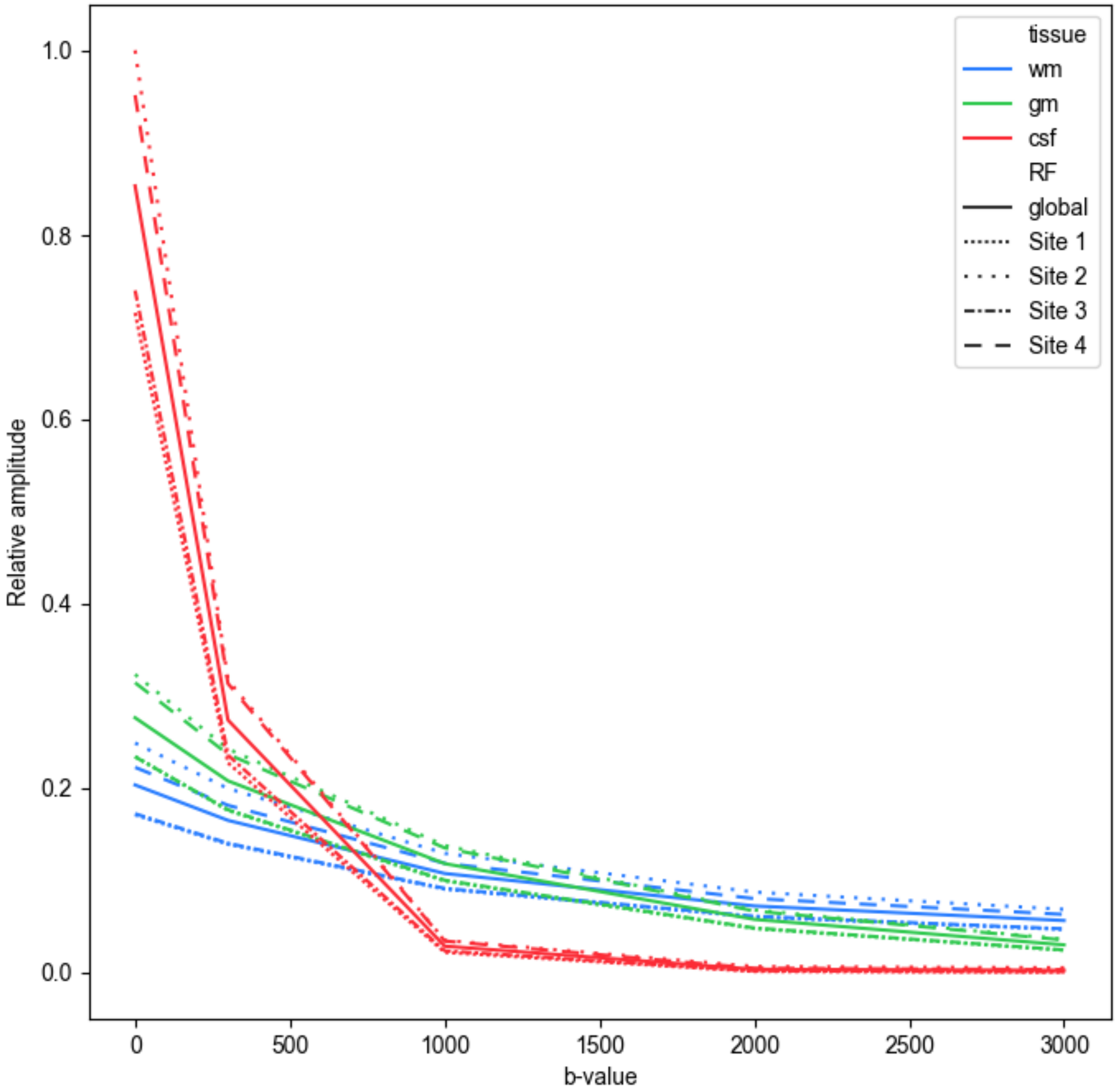

Supplementary Figure S1: Response function (RF) estimations. Line plot shows the average relative signal amplitude for response functions for each tissue type as a function of b-value, for each of the different response functions included in the study (site-specific RFs and global RF).

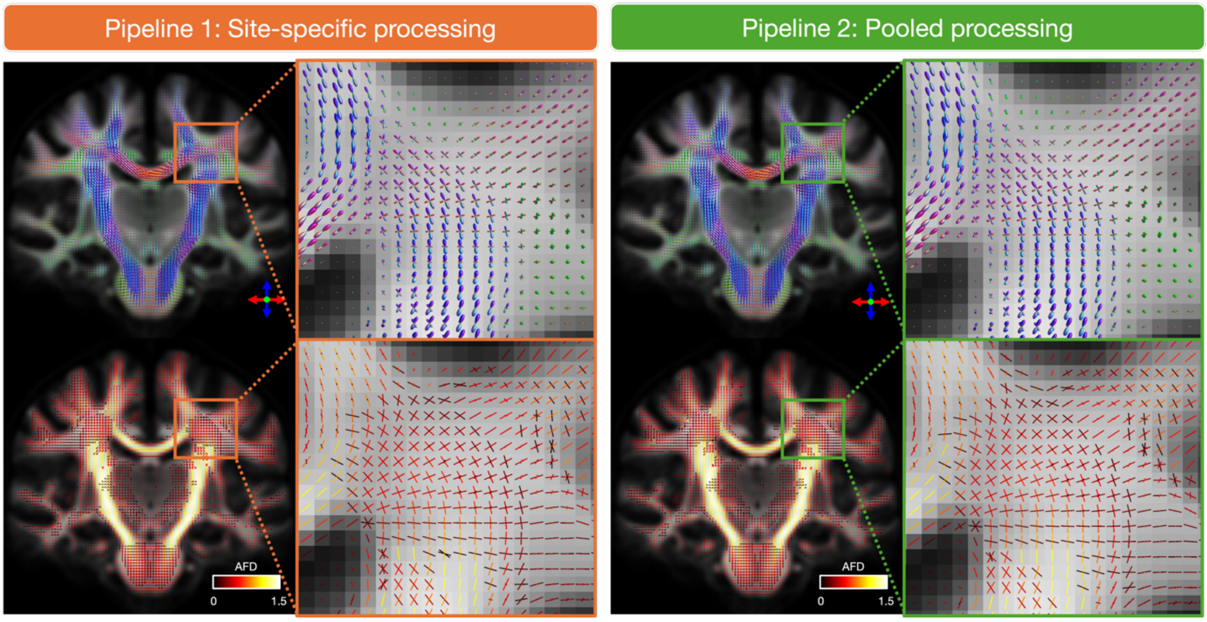

Supplementary Figure S2: Comparison between Pipelines 1 & 2. The inclusion of two pipelines resulted in two FOD templates. The left images show outputs from Pipeline 1 (site-specific processing), while right images show outputs from Pipeline 2 (pooled processing). Upon visual inspection, these two templates were highly similar, with no obvious appreciable differences. The top images show the FODs overlaid on the template image, while bottom images show the template fixels coloured by the template apparent fibre density (AFD) value (derived from segmentation of the template FODs).

Table S1: Comparison between Pipelines 1 & 2

Pipeline	Count	AFD				
		Mean	Median	SD	Min	Max
Pipeline 1 (site-specific)	627896	0.332	0.274	0.217	0.0003	1.437
Pipeline 2 (pooled)	625296	0.329	0.271	0.217	0.0003	1.441

Additional results for tract-level analyses (Supplement to Section 3.2.2)

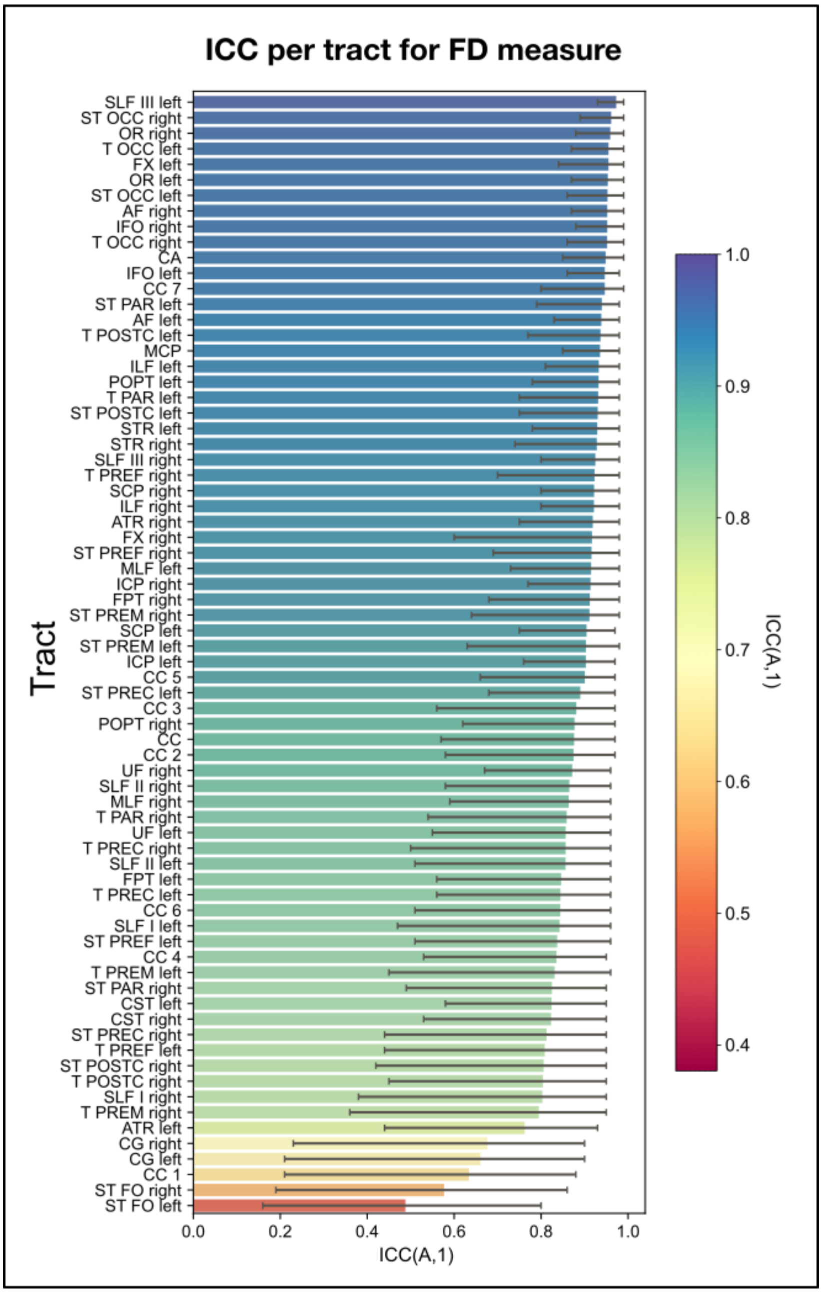

Supplementary Figure S3: Intraclass correlation coefficient (ICC(A,1)) for the FD measure per tract for Pipeline 2. Bar plot shows ICC for each tract (ordered from highest ICC to lowest). FD values were computed using Pipeline 2 (group-based processing), which yielded similar reproducibility results to Pipeline 1 (data shown in Figure 3). Bars are coloured by the ICC value, with the same colour scale used here as for Figure 3 and Figure 6 for comparability. Grey bars show the 95% confidence interval for ICC(A,1).

Table S2: Summary metrics of tract-level reproducibility and reliability

Pipeline	Metric	CV_{ws} (%)			ICC(A,1)		
		Mean	SD	Range	Mean	SD	Range
Pipeline 1 (site-specific)	FD	1.48	0.54	[0.74 – 3.63]	0.859	0.084	[0.538 – 0.963]
	FC	1.06	0.32	[0.54 – 1.87]	0.980	0.013	[0.939 – 0.996]
	FDC	2.24	0.73	[1.23 – 5.03]	0.946	0.034	[0.813 – 0.989]
Pipeline 2 (pooled)	FD	1.41	0.63	[0.78 – 4.01]	0.873	0.091	[0.488 – 0.973]
	FC	1.00	0.28	[0.60 – 1.76]	0.982	0.011	[0.945 – 0.997]
	FDC	2.11	0.76	[1.22 – 5.66]	0.952	0.034	[0.829 – 0.989]

Table S3: CV and ICC of FD at the tract level (FD computed using site-specific response functions)

Tract	mean	std	CV_{ws}	CV_{bs}	ICC(A,1)	ICC 95% CI	p-value
**AF left**	0.355	0.014	1.11	3.96	0.925	[0.75, 0.98]	0.000000
**AF right**	0.355	0.015	1.17	4.13	0.924	[0.73, 0.98]	0.000000
**ATR left**	0.325	0.013	2.00	3.62	0.764	[0.43, 0.93]	0.000000
**ATR right**	0.330	0.016	1.48	4.90	0.914	[0.71, 0.98]	0.000000
**CA**	0.342	0.032	2.46	9.38	0.933	[0.78, 0.98]	0.000000
**CC 1**	0.408	0.024	3.63	5.29	0.677	[0.27, 0.9]	0.000000
**CC 2**	0.390	0.016	1.44	4.13	0.892	[0.64, 0.97]	0.000000
**CC 3**	0.404	0.025	2.20	6.23	0.889	[0.59, 0.97]	0.000000
**CC 4**	0.512	0.015	1.51	2.71	0.761	[0.33, 0.93]	0.000000
**CC 5**	0.471	0.016	1.48	3.26	0.830	[0.41, 0.96]	0.000000
**CC 6**	0.434	0.010	0.96	2.21	0.838	[0.49, 0.96]	0.000000
**CC 7**	0.400	0.015	0.74	3.84	0.963	[0.91, 0.99]	0.000000
**CC**	0.428	0.012	1.13	2.79	0.858	[0.51, 0.96]	0.000000
**CG left**	0.322	0.011	1.94	3.14	0.716	[0.28, 0.92]	0.000000
**CG right**	0.313	0.011	1.88	3.24	0.738	[0.33, 0.92]	0.000000
**CST left**	0.575	0.015	1.43	2.38	0.733	[0.33, 0.92]	0.000000
**CST right**	0.584	0.016	1.28	2.60	0.802	[0.44, 0.95]	0.000000
**FPT left**	0.481	0.014	1.20	2.88	0.852	[0.57, 0.96]	0.000000
**FPT right**	0.488	0.018	1.16	3.75	0.913	[0.67, 0.98]	0.000000
**FX left**	0.490	0.032	1.65	6.63	0.942	[0.76, 0.99]	0.000000
**FX right**	0.451	0.032	2.24	7.05	0.908	[0.55, 0.98]	0.000000
**ICP left**	0.359	0.013	1.39	3.69	0.874	[0.67, 0.96]	0.000000
**ICP right**	0.338	0.015	1.43	4.58	0.911	[0.76, 0.97]	0.000000
**IFO left**	0.378	0.017	1.10	4.58	0.944	[0.86, 0.98]	0.000000
**IFO right**	0.362	0.015	0.98	4.14	0.945	[0.85, 0.98]	0.000000
**ILF left**	0.367	0.015	1.01	4.08	0.939	[0.85, 0.98]	0.000000
**ILF right**	0.357	0.012	1.21	3.32	0.880	[0.61, 0.97]	0.000000
**MCP**	0.374	0.015	1.17	4.01	0.920	[0.8, 0.98]	0.000000
**MLF left**	0.393	0.011	0.91	2.92	0.909	[0.73, 0.98]	0.000000
**MLF right**	0.399	0.012	1.29	2.78	0.818	[0.41, 0.95]	0.000000
**OR left**	0.381	0.017	1.00	4.47	0.950	[0.86, 0.99]	0.000000
**OR right**	0.382	0.014	0.98	3.82	0.937	[0.79, 0.98]	0.000000
**POPT left**	0.488	0.018	1.20	3.63	0.900	[0.63, 0.97]	0.000000
**POPT right**	0.499	0.015	1.24	2.92	0.847	[0.5, 0.96]	0.000000
**SCP left**	0.400	0.014	1.01	3.43	0.919	[0.81, 0.98]	0.000000
**SCP right**	0.367	0.016	1.12	4.54	0.941	[0.86, 0.98]	0.000000
**SLF III left**	0.353	0.018	1.08	5.29	0.961	[0.88, 0.99]	0.000000
**SLF III right**	0.346	0.010	1.25	2.75	0.825	[0.43, 0.95]	0.000000

**SLF II left**	0.337	0.020	2.36	5.75	0.853	[0.51, 0.96]	0.000000
**SLF II right**	0.348	0.014	1.54	3.79	0.854	[0.51, 0.96]	0.000000
**SLF I left**	0.325	0.016	2.01	4.83	0.850	[0.48, 0.96]	0.000000
**SLF I right**	0.338	0.015	2.02	4.12	0.803	[0.35, 0.95]	0.000000
**ST FO left**	0.318	0.014	3.09	3.59	0.538	[0.21, 0.83]	0.000007
**ST FO right**	0.327	0.015	2.89	3.82	0.618	[0.25, 0.87]	0.000000
**ST OCC left**	0.394	0.017	0.98	4.45	0.953	[0.87, 0.99]	0.000000
**ST OCC right**	0.390	0.015	1.04	3.83	0.929	[0.74, 0.98]	0.000000
**ST PAR left**	0.432	0.015	1.04	3.48	0.915	[0.65, 0.98]	0.000000
**ST PAR right**	0.438	0.011	1.24	2.25	0.763	[0.31, 0.94]	0.000000
**ST POSTC left**	0.444	0.017	1.37	3.73	0.878	[0.49, 0.97]	0.000000
**ST POSTC right**	0.463	0.012	1.37	2.38	0.748	[0.27, 0.93]	0.000000
**ST PREC left**	0.457	0.013	1.31	2.76	0.814	[0.4, 0.95]	0.000000
**ST PREC right**	0.476	0.012	1.28	2.28	0.756	[0.3, 0.93]	0.000000
**ST PREF left**	0.371	0.013	1.39	3.33	0.850	[0.55, 0.96]	0.000000
**ST PREF right**	0.375	0.017	1.27	4.64	0.930	[0.75, 0.98]	0.000000
**ST PREM left**	0.362	0.019	1.72	5.21	0.902	[0.64, 0.98]	0.000000
**ST PREM right**	0.403	0.025	1.90	6.16	0.913	[0.64, 0.98]	0.000000
**STR left**	0.577	0.023	1.43	3.94	0.885	[0.54, 0.97]	0.000000
**STR right**	0.582	0.021	1.07	3.62	0.920	[0.73, 0.98]	0.000000
**T OCC left**	0.380	0.017	0.99	4.46	0.952	[0.86, 0.99]	0.000000
**T OCC right**	0.382	0.014	0.96	3.59	0.932	[0.78, 0.98]	0.000000
**T PAR left**	0.427	0.015	1.09	3.56	0.911	[0.65, 0.98]	0.000000
**T PAR right**	0.440	0.013	1.23	2.82	0.839	[0.45, 0.96]	0.000000
**T POSTC left**	0.450	0.018	1.37	3.89	0.887	[0.54, 0.97]	0.000000
**T POSTC right**	0.470	0.012	1.31	2.27	0.746	[0.3, 0.93]	0.000000
**T PREC left**	0.479	0.013	1.39	2.58	0.774	[0.35, 0.94]	0.000000
**T PREC right**	0.498	0.015	1.26	2.81	0.832	[0.43, 0.96]	0.000000
**T PREF left**	0.389	0.013	1.46	3.11	0.818	[0.46, 0.95]	0.000000
**T PREF right**	0.381	0.018	1.28	4.66	0.930	[0.72, 0.98]	0.000000
**T PREM left**	0.372	0.017	1.93	4.46	0.842	[0.49, 0.96]	0.000000
**T PREM right**	0.405	0.018	2.04	4.23	0.812	[0.39, 0.95]	0.000000
**UF left**	0.346	0.017	2.21	4.81	0.820	[0.44, 0.95]	0.000000
**UF right**	0.335	0.019	2.12	5.49	0.869	[0.65, 0.96]	0.000000

Table S4: CV and ICC of FC at the tract level (FC computed using site-specific response functions)

Tract	mean	std	CV_{ws}	CV_{bs}	ICC(A,1)	ICC 95% CI	p-value
**AF left**	1.046	0.080	0.80	7.91	0.990	[0.97, 1.0]	0.000000
**AF right**	1.051	0.079	0.86	7.77	0.988	[0.95, 1.0]	0.000000
**ATR left**	1.030	0.068	0.90	6.83	0.983	[0.92, 1.0]	0.000000
**ATR right**	1.031	0.069	0.91	6.92	0.983	[0.93, 1.0]	0.000000
**CA**	1.037	0.099	0.94	9.91	0.992	[0.98, 1.0]	0.000000
**CC 1**	1.065	0.103	1.53	9.93	0.974	[0.92, 0.99]	0.000000
**CC 2**	1.039	0.070	1.14	6.93	0.973	[0.89, 0.99]	0.000000
**CC 3**	1.033	0.077	1.49	7.65	0.964	[0.87, 0.99]	0.000000
**CC 4**	1.030	0.092	1.87	9.14	0.960	[0.86, 0.99]	0.000000
**CC 5**	1.013	0.069	1.80	6.95	0.939	[0.8, 0.98]	0.000000
**CC 6**	1.021	0.074	0.80	7.47	0.989	[0.97, 1.0]	0.000000
**CC 7**	1.121	0.116	0.84	10.74	0.994	[0.97, 1.0]	0.000000
**CC**	1.033	0.070	0.98	7.01	0.981	[0.94, 1.0]	0.000000
**CG left**	1.024	0.072	0.93	7.28	0.984	[0.95, 1.0]	0.000000
**CG right**	1.023	0.078	1.11	7.84	0.980	[0.92, 1.0]	0.000000
**CST left**	1.019	0.087	1.30	8.78	0.979	[0.91, 0.99]	0.000000
**CST right**	1.024	0.080	1.37	8.07	0.971	[0.86, 0.99]	0.000000
**FPT left**	1.018	0.069	0.93	7.03	0.983	[0.92, 1.0]	0.000000
**FPT right**	1.023	0.072	0.94	7.27	0.984	[0.91, 1.0]	0.000000
**FX left**	0.971	0.074	1.25	7.82	0.974	[0.91, 0.99]	0.000000
**FX right**	0.978	0.087	1.00	9.20	0.988	[0.96, 1.0]	0.000000
**ICP left**	1.050	0.057	0.75	5.60	0.982	[0.95, 1.0]	0.000000
**ICP right**	1.053	0.058	0.99	5.62	0.970	[0.92, 0.99]	0.000000
**IFO left**	1.103	0.103	0.73	9.67	0.994	[0.97, 1.0]	0.000000
**IFO right**	1.106	0.102	0.59	9.63	0.996	[0.98, 1.0]	0.000000
**ILF left**	1.119	0.117	0.79	10.87	0.995	[0.98, 1.0]	0.000000
**ILF right**	1.121	0.118	0.83	10.90	0.994	[0.97, 1.0]	0.000000
**MCP**	1.069	0.057	0.66	5.50	0.986	[0.97, 1.0]	0.000000
**MLF left**	1.035	0.082	0.74	8.22	0.993	[0.98, 1.0]	0.000000
**MLF right**	1.033	0.093	0.77	9.34	0.993	[0.98, 1.0]	0.000000
**OR left**	1.111	0.121	0.75	11.27	0.995	[0.98, 1.0]	0.000000
**OR right**	1.117	0.119	0.77	11.07	0.995	[0.98, 1.0]	0.000000
**POPT left**	1.000	0.067	0.88	6.88	0.984	[0.96, 1.0]	0.000000
**POPT right**	1.002	0.080	0.82	8.29	0.991	[0.97, 1.0]	0.000000
**SCP left**	1.037	0.064	0.54	6.44	0.993	[0.98, 1.0]	0.000000
**SCP right**	1.034	0.061	0.71	6.08	0.987	[0.97, 1.0]	0.000000
**SLF III left**	1.045	0.081	0.90	7.98	0.988	[0.96, 1.0]	0.000000
**SLF III right**	1.048	0.073	0.93	7.17	0.983	[0.94, 1.0]	0.000000

**SLF II left**	1.038	0.084	1.18	8.39	0.981	[0.94, 1.0]	0.000000
**SLF II right**	1.042	0.075	1.09	7.46	0.979	[0.92, 0.99]	0.000000
**SLF I left**	1.023	0.087	1.63	8.77	0.966	[0.89, 0.99]	0.000000
**SLF I right**	1.027	0.084	1.39	8.38	0.974	[0.9, 0.99]	0.000000
**ST FO left**	1.052	0.086	0.83	8.45	0.990	[0.97, 1.0]	0.000000
**ST FO right**	1.058	0.086	0.99	8.44	0.986	[0.96, 1.0]	0.000000
**ST OCC left**	1.114	0.121	0.76	11.32	0.995	[0.98, 1.0]	0.000000
**ST OCC right**	1.118	0.114	0.78	10.63	0.994	[0.97, 1.0]	0.000000
**ST PAR left**	1.010	0.065	0.91	6.64	0.982	[0.95, 1.0]	0.000000
**ST PAR right**	1.013	0.069	0.89	7.05	0.985	[0.96, 1.0]	0.000000
**ST POSTC left**	1.000	0.062	1.37	6.34	0.957	[0.86, 0.99]	0.000000
**ST POSTC right**	0.998	0.058	1.42	5.86	0.945	[0.82, 0.99]	0.000000
**ST PREC left**	1.018	0.075	1.49	7.55	0.963	[0.88, 0.99]	0.000000
**ST PREC right**	1.021	0.076	1.57	7.66	0.960	[0.85, 0.99]	0.000000
**ST PREF left**	1.034	0.065	1.00	6.53	0.977	[0.91, 0.99]	0.000000
**ST PREF right**	1.040	0.070	1.00	6.96	0.979	[0.91, 1.0]	0.000000
**ST PREM left**	1.022	0.080	1.26	8.02	0.976	[0.92, 0.99]	0.000000
**ST PREM right**	1.023	0.058	1.22	5.76	0.958	[0.85, 0.99]	0.000000
**STR left**	1.027	0.093	1.82	9.32	0.965	[0.88, 0.99]	0.000000
**STR right**	1.019	0.080	1.66	8.03	0.960	[0.85, 0.99]	0.000000
**T OCC left**	1.108	0.119	0.75	11.13	0.995	[0.98, 1.0]	0.000000
**T OCC right**	1.112	0.115	0.77	10.76	0.995	[0.98, 1.0]	0.000000
**T PAR left**	1.009	0.065	0.93	6.61	0.981	[0.95, 0.99]	0.000000
**T PAR right**	1.006	0.070	0.87	7.24	0.986	[0.96, 1.0]	0.000000
**T POSTC left**	1.002	0.066	1.31	6.78	0.965	[0.89, 0.99]	0.000000
**T POSTC right**	0.997	0.060	1.29	6.18	0.959	[0.86, 0.99]	0.000000
**T PREC left**	1.019	0.081	1.50	8.20	0.968	[0.89, 0.99]	0.000000
**T PREC right**	1.021	0.081	1.48	8.18	0.968	[0.88, 0.99]	0.000000
**T PREF left**	1.026	0.065	1.07	6.51	0.974	[0.9, 0.99]	0.000000
**T PREF right**	1.031	0.070	1.06	7.04	0.978	[0.9, 0.99]	0.000000
**T PREM left**	1.018	0.078	1.21	7.90	0.977	[0.92, 0.99]	0.000000
**T PREM right**	1.015	0.064	1.20	6.49	0.967	[0.88, 0.99]	0.000000
**UF left**	1.067	0.116	1.01	11.32	0.992	[0.96, 1.0]	0.000000
**UF right**	1.084	0.111	0.69	10.59	0.996	[0.99, 1.0]	0.000000

Table S5: CV and ICC of FDC at the tract level (FDC computed using site-specific response functions)

Tract	mean	std	CV_{ws}	CV_{bs}	ICC(A,1)	ICC 95% CI	p-value
**AF left**	0.374	0.040	1.57	11.00	0.979	[0.93, 0.99]	0.000000
**AF right**	0.376	0.039	1.59	10.63	0.977	[0.91, 0.99]	0.000000
**ATR left**	0.337	0.028	2.19	8.41	0.938	[0.8, 0.98]	0.000000
**ATR right**	0.342	0.035	1.78	10.55	0.971	[0.9, 0.99]	0.000000
**CA**	0.356	0.065	3.31	18.62	0.969	[0.9, 0.99]	0.000000
**CC 1**	0.431	0.058	5.03	13.17	0.870	[0.59, 0.97]	0.000000
**CC 2**	0.406	0.037	2.08	9.29	0.952	[0.82, 0.99]	0.000000
**CC 3**	0.420	0.045	3.28	10.77	0.916	[0.7, 0.98]	0.000000
**CC 4**	0.526	0.054	3.26	10.25	0.908	[0.66, 0.98]	0.000000
**CC 5**	0.477	0.042	3.17	8.67	0.883	[0.58, 0.97]	0.000000
**CC 6**	0.445	0.038	1.35	8.72	0.976	[0.92, 0.99]	0.000000
**CC 7**	0.443	0.049	1.32	11.50	0.987	[0.95, 1.0]	0.000000
**CC**	0.443	0.036	1.85	8.32	0.951	[0.83, 0.99]	0.000000
**CG left**	0.330	0.031	2.26	9.58	0.945	[0.8, 0.99]	0.000000
**CG right**	0.322	0.028	2.30	8.80	0.935	[0.72, 0.98]	0.000000
**CST left**	0.585	0.057	2.72	9.88	0.930	[0.71, 0.98]	0.000000
**CST right**	0.596	0.054	2.63	9.07	0.921	[0.67, 0.98]	0.000000
**FPT left**	0.489	0.042	1.92	8.68	0.952	[0.83, 0.99]	0.000000
**FPT right**	0.499	0.047	1.73	9.77	0.969	[0.86, 0.99]	0.000000
**FX left**	0.472	0.040	2.18	8.63	0.940	[0.79, 0.98]	0.000000
**FX right**	0.436	0.028	2.84	6.12	0.813	[0.41, 0.95]	0.000000
**ICP left**	0.375	0.028	1.73	7.48	0.950	[0.86, 0.99]	0.000000
**ICP right**	0.354	0.029	1.95	8.48	0.950	[0.87, 0.99]	0.000000
**IFO left**	0.412	0.047	1.43	11.72	0.985	[0.96, 1.0]	0.000000
**IFO right**	0.396	0.043	1.29	11.12	0.986	[0.96, 1.0]	0.000000
**ILF left**	0.409	0.053	1.47	13.52	0.987	[0.96, 1.0]	0.000000
**ILF right**	0.395	0.046	1.88	12.01	0.976	[0.87, 0.99]	0.000000
**MCP**	0.400	0.031	1.54	8.04	0.965	[0.91, 0.99]	0.000000
**MLF left**	0.407	0.038	1.25	9.65	0.983	[0.95, 1.0]	0.000000
**MLF right**	0.413	0.043	1.71	10.61	0.974	[0.88, 0.99]	0.000000
**OR left**	0.418	0.056	1.48	13.96	0.989	[0.96, 1.0]	0.000000
**OR right**	0.422	0.045	1.53	11.09	0.981	[0.92, 1.0]	0.000000
**POPT left**	0.490	0.044	1.95	9.19	0.956	[0.82, 0.99]	0.000000
**POPT right**	0.501	0.044	1.96	8.96	0.954	[0.81, 0.99]	0.000000
**SCP left**	0.411	0.038	1.23	9.52	0.984	[0.96, 1.0]	0.000000
**SCP right**	0.376	0.038	1.51	10.30	0.979	[0.95, 0.99]	0.000000
**SLF III left**	0.372	0.037	1.55	10.37	0.977	[0.93, 0.99]	0.000000
**SLF III right**	0.364	0.031	1.81	8.79	0.957	[0.81, 0.99]	0.000000

**SLF II left**	0.354	0.042	3.04	12.05	0.936	[0.77, 0.98]	0.000000
**SLF II right**	0.367	0.040	2.26	11.03	0.958	[0.83, 0.99]	0.000000
**SLF I left**	0.337	0.039	3.20	11.72	0.928	[0.74, 0.98]	0.000000
**SLF I right**	0.351	0.041	3.06	11.91	0.938	[0.73, 0.98]	0.000000
**ST FO left**	0.335	0.035	3.53	10.50	0.887	[0.72, 0.97]	0.000000
**ST FO right**	0.347	0.038	3.73	11.00	0.894	[0.7, 0.97]	0.000000
**ST OCC left**	0.431	0.055	1.49	13.21	0.987	[0.95, 1.0]	0.000000
**ST OCC right**	0.428	0.045	1.62	10.78	0.977	[0.88, 0.99]	0.000000
**ST PAR left**	0.437	0.037	1.66	8.59	0.963	[0.86, 0.99]	0.000000
**ST PAR right**	0.444	0.035	1.95	8.09	0.944	[0.77, 0.99]	0.000000
**ST POSTC left**	0.444	0.040	2.68	9.15	0.921	[0.66, 0.98]	0.000000
**ST POSTC right**	0.461	0.035	2.85	7.49	0.875	[0.53, 0.97]	0.000000
**ST PREC left**	0.465	0.043	2.74	9.21	0.919	[0.68, 0.98]	0.000000
**ST PREC right**	0.485	0.043	2.84	8.86	0.907	[0.62, 0.98]	0.000000
**ST PREF left**	0.384	0.030	1.91	8.00	0.945	[0.82, 0.99]	0.000000
**ST PREF right**	0.391	0.038	1.82	10.05	0.967	[0.86, 0.99]	0.000000
**ST PREM left**	0.374	0.039	2.46	10.62	0.948	[0.81, 0.99]	0.000000
**ST PREM right**	0.414	0.036	2.76	8.58	0.907	[0.66, 0.98]	0.000000
**STR left**	0.592	0.072	3.39	12.38	0.934	[0.72, 0.98]	0.000000
**STR right**	0.594	0.063	2.83	10.79	0.939	[0.73, 0.99]	0.000000
**T OCC left**	0.416	0.055	1.47	13.68	0.988	[0.95, 1.0]	0.000000
**T OCC right**	0.420	0.044	1.50	10.87	0.981	[0.92, 1.0]	0.000000
**T PAR left**	0.432	0.037	1.73	8.69	0.961	[0.85, 0.99]	0.000000
**T PAR right**	0.444	0.036	1.90	8.18	0.948	[0.79, 0.99]	0.000000
**T POSTC left**	0.452	0.043	2.64	9.55	0.928	[0.69, 0.98]	0.000000
**T POSTC right**	0.467	0.038	2.65	8.19	0.904	[0.62, 0.98]	0.000000
**T PREC left**	0.489	0.048	2.84	9.79	0.922	[0.69, 0.98]	0.000000
**T PREC right**	0.508	0.048	2.76	9.60	0.923	[0.68, 0.98]	0.000000
**T PREF left**	0.400	0.031	2.06	7.74	0.932	[0.78, 0.98]	0.000000
**T PREF right**	0.394	0.039	1.87	10.27	0.967	[0.85, 0.99]	0.000000
**T PREM left**	0.382	0.038	2.63	10.11	0.934	[0.76, 0.98]	0.000000
**T PREM right**	0.414	0.036	2.82	8.60	0.905	[0.65, 0.98]	0.000000
**UF left**	0.368	0.056	2.81	15.69	0.966	[0.85, 0.99]	0.000000
**UF right**	0.361	0.054	2.51	15.42	0.974	[0.92, 0.99]	0.000000

Additional results for fixel-level analyses (Supplement to Section 3.2.3)

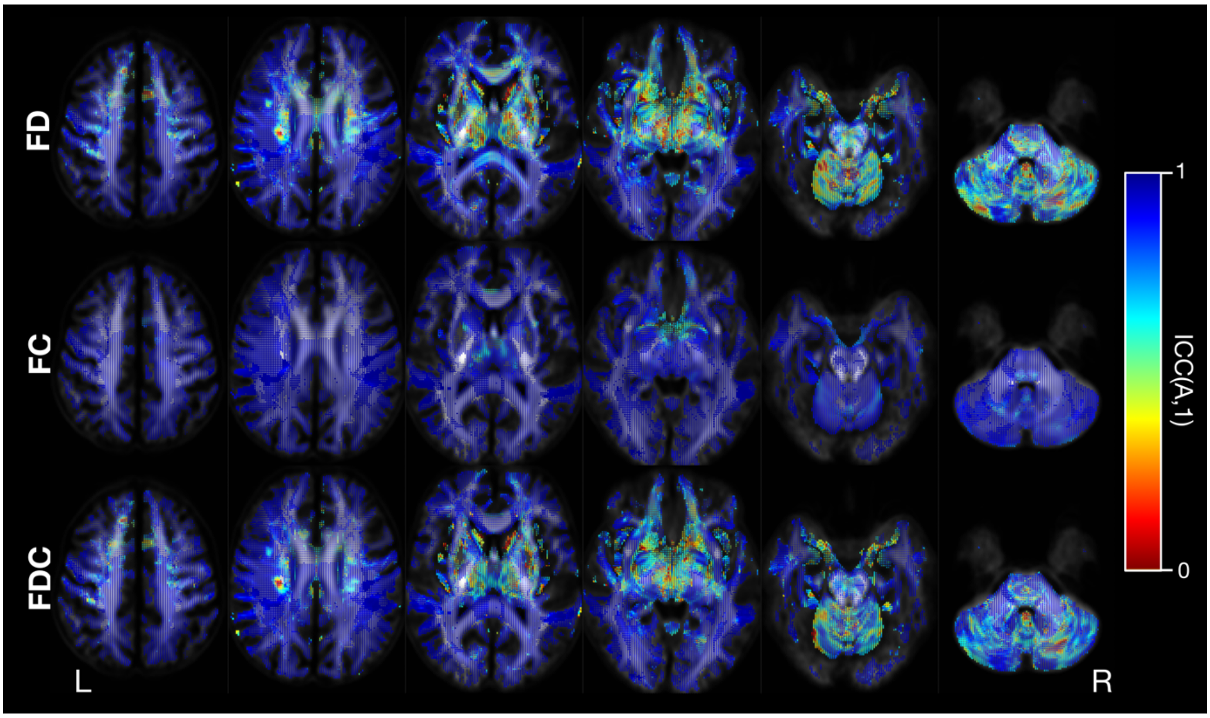

Supplementary Figure S4: Intraclass correlation coefficient (ICC(A,1)) for fixel-based metrics per fixel for Pipeline 2. Top row shows ICC for the fibre density (FD) metric, middle row shows ICC for the fibre cross-section (FC) metric, and bottom row shows ICC for the fibre density and cross-section (FDC) metric. Results were very comparable to Pipeline 1 (Figure 4).

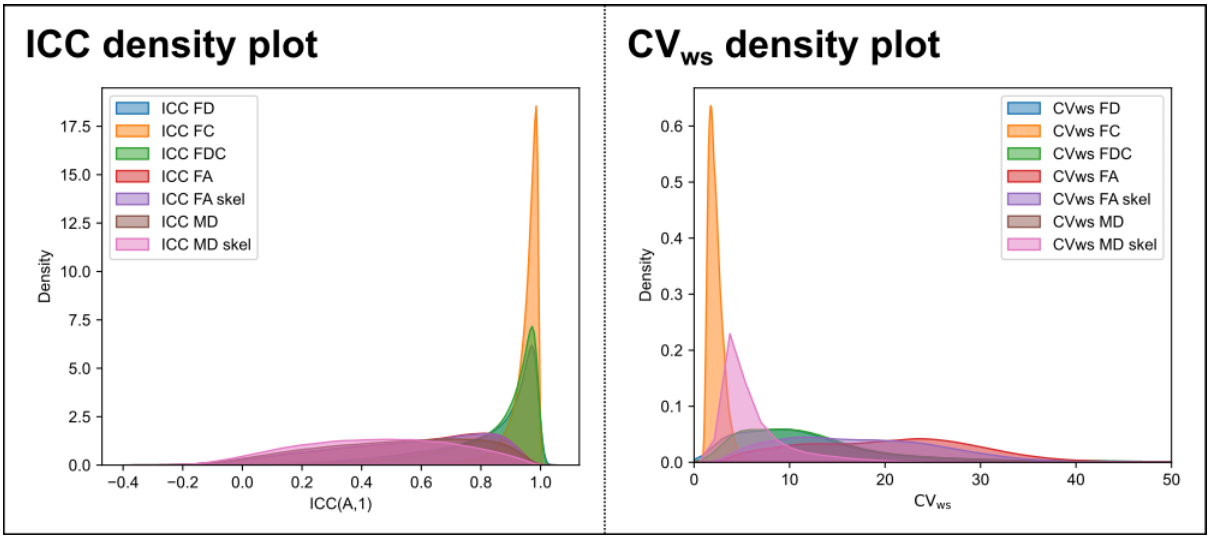

Supplementary Figure S5: Density plots for ICC and CV_{ws} at each fixel/voxel. The left plot shows distribution of ICC values across all fixels (for FBA metrics) and voxels (for DTI metrics) across the brain white matter. The right plot shows distribution density of CV_{ws} across brain white matter. For the FBA metrics, included fixels were defined on the FOD template image. For the DTI metrics, we used a voxel mask that corresponded to the fixel mask (including all voxels with containing white matter fixels). However, we additionally computed a constrained WM skeleton mask using an FA threshold of FA > 0.2. This data is summarised in Table 4.

Additional results for tensor-based metrics (Supplement to Section 3.3)

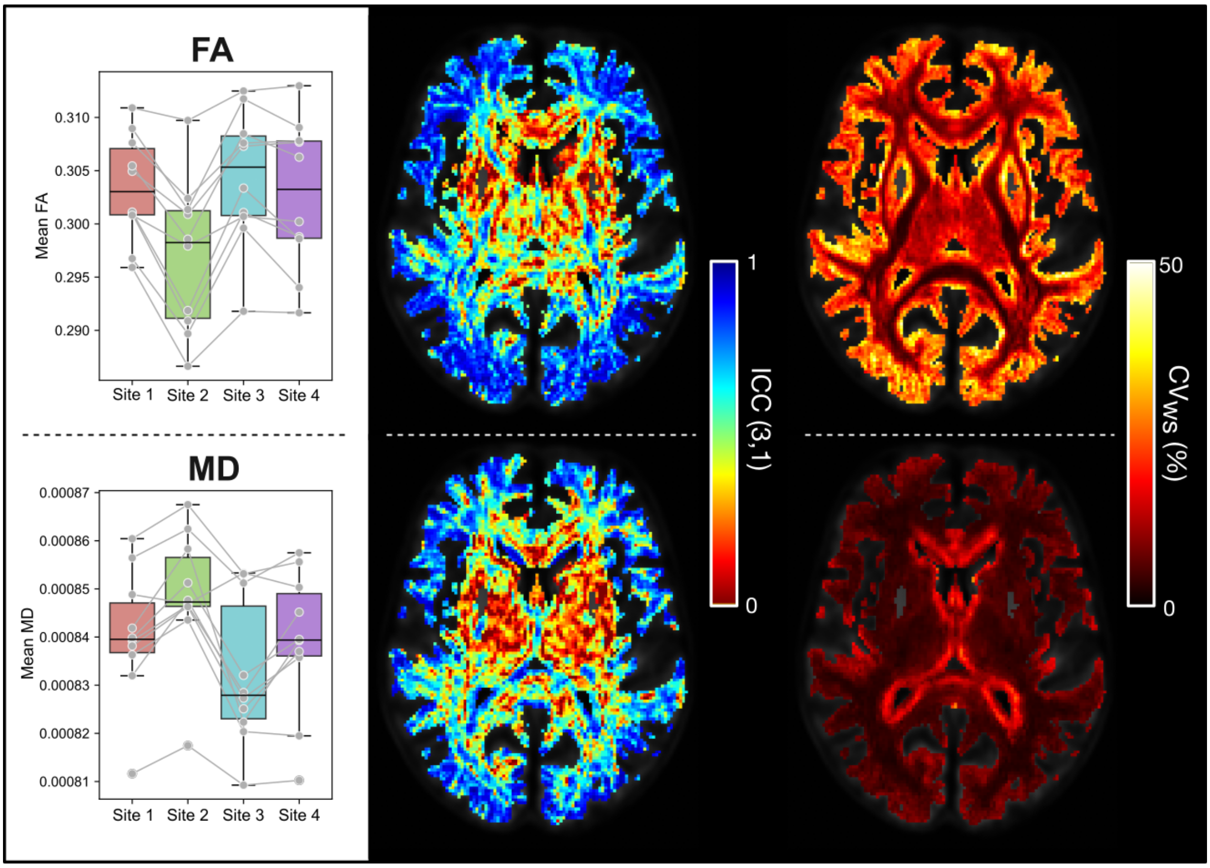

Supplementary Figure S6: Reliability and reproducibility of tensor-based measures. Top panel shows results for fractional anisotropy (FA), and bottom panel shows results for mean diffusivity (MD). Boxplots on the left show mean values across the whole brain, constrained to a white matter fixel mask (converted to a voxel mask). Middle images show ICC value at each voxel in the white matter voxel mask for a single axial slice. ICC values were generally lower in subcortical and cerebellar regions. Right images show CV_{ws} (%) at each voxel. The FA measure was generally more reproducible in key white matter regions, with higher variability towards cortical regions. MD had high reproducibility throughout the brain, with voxels surrounding the ventricles exhibiting slightly higher CV_{ws}.

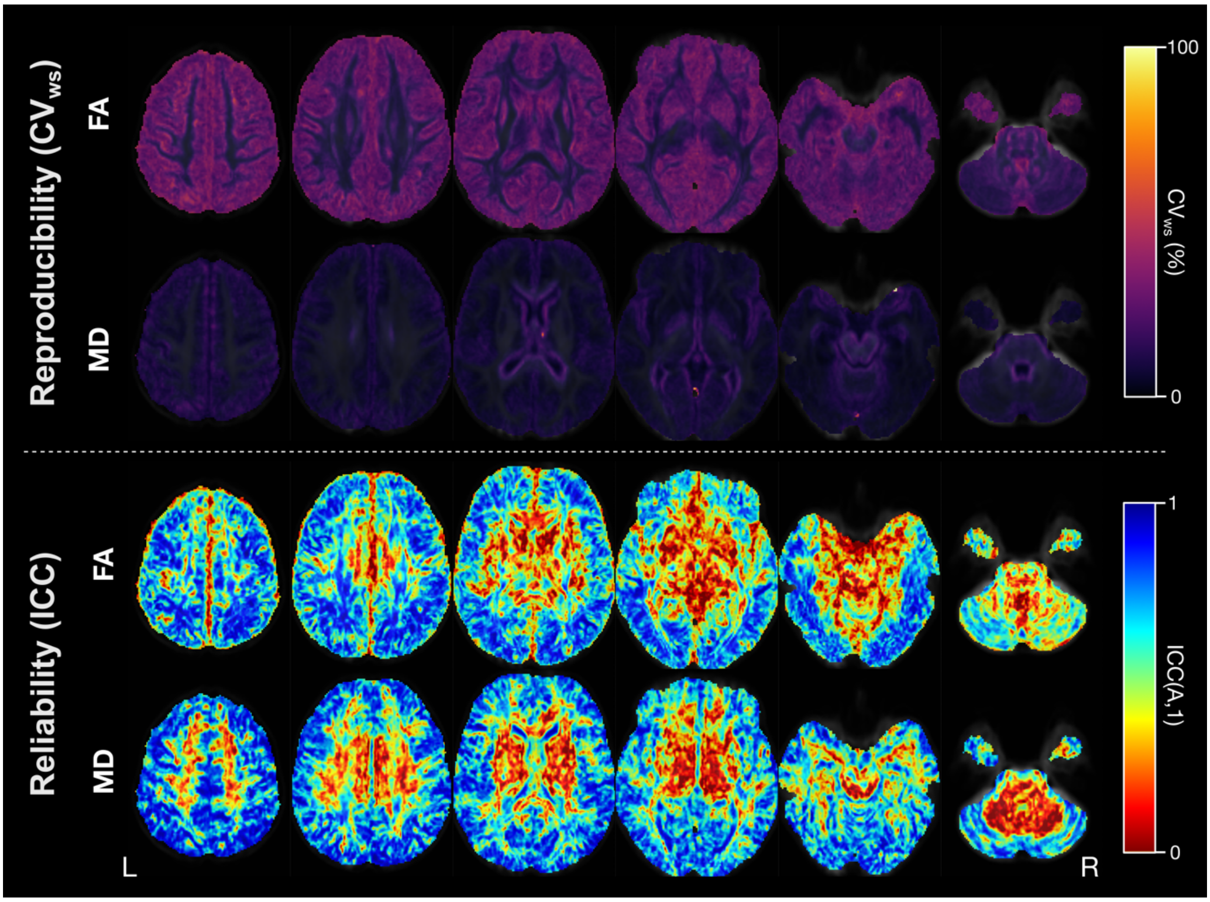

Supplementary Figure S7: Reproducibility and reliability of tensor-based measures across the whole brain. For transparency, we additionally provide the reproducibility and reliability results for DTI-based metrics across the whole brain (not constrained to white matter). Top panel shows reproducibility (CV_{ws}) results for fractional anisotropy (FA) and mean diffusivity (MD). Bottom panel shows reliability (ICC) results FA and MD.

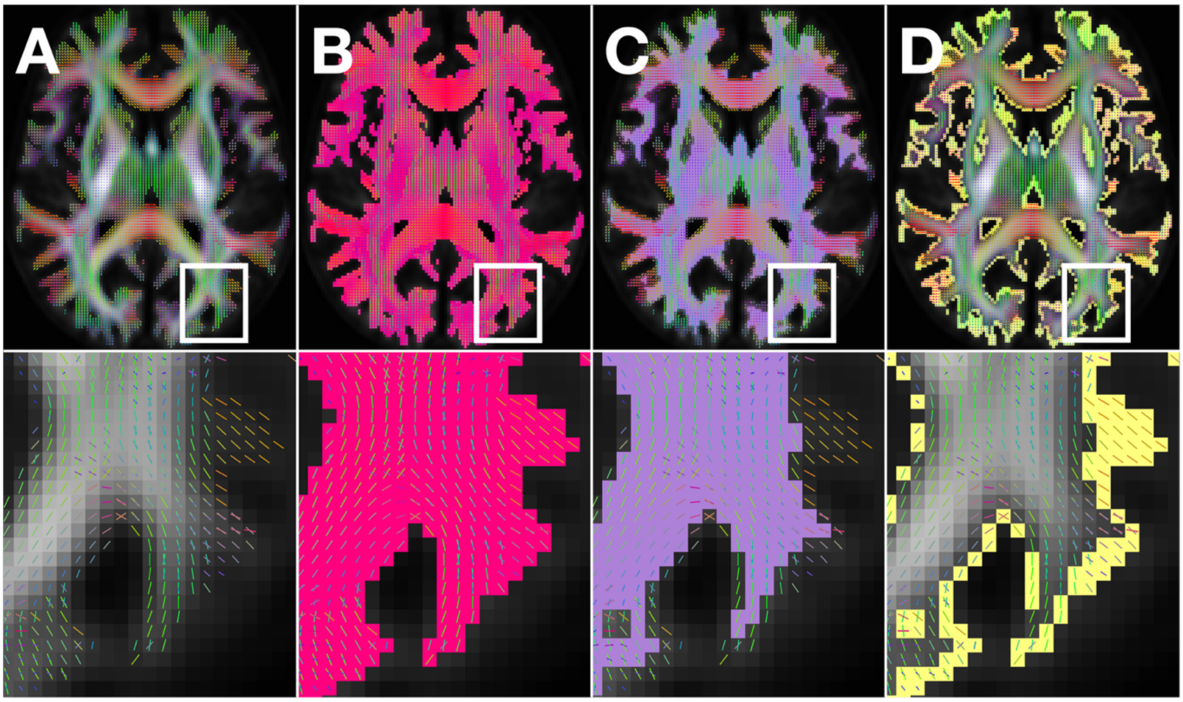

Supplementary Figure S8: Fixel and voxel masks used for analysis. Top rows show a single axial slice of the template image used for both FBA and DTI analyses, and bottom row shows a zoomed inset. The fixel mask (fixels included in the analysis) are shown on Panel A (as well as across all panels). The voxel mask used for the DTI analysis is shown in Panel B, and included all voxels containing at least one fixel. We additionally computed a constrained voxel mask for the purposes of DTI analyses (Panel C), and the difference between the two voxel masks is shown in Panel D.

Table S6: Reproducibility and reliability of FA at the tract level

Tract	mean	std	CV_{ws}	CV_{bs}	ICC(A,1)	ICC 95% CI	p-value
**AF left**	0.347	0.011	1.99	2.91	0.678	[0.22, 0.9]	0.000000
**AF right**	0.354	0.012	1.97	2.93	0.683	[0.24, 0.91]	0.000000
**ATR left**	0.320	0.015	2.90	4.10	0.664	[0.22, 0.9]	0.000000
**ATR right**	0.324	0.014	1.81	4.34	0.851	[0.48, 0.96]	0.000000
**CA**	0.340	0.024	2.75	7.00	0.858	[0.53, 0.96]	0.000000
**CC 1**	0.434	0.021	1.94	4.82	0.857	[0.68, 0.96]	0.000000
**CC 2**	0.357	0.014	3.12	3.07	0.485	[0.09, 0.81]	0.000000
**CC 3**	0.364	0.021	4.58	4.23	0.454	[0.08, 0.8]	0.000000
**CC 4**	0.404	0.011	2.32	2.08	0.429	[0.07, 0.78]	0.000000
**CC 5**	0.394	0.012	1.76	2.60	0.678	[0.26, 0.9]	0.000000
**CC 6**	0.383	0.006	0.65	1.48	0.832	[0.6, 0.95]	0.000000
**CC 7**	0.355	0.008	0.77	2.33	0.902	[0.73, 0.97]	0.000000
**CC**	0.382	0.009	1.75	2.01	0.561	[0.13, 0.86]	0.000000
**CG left**	0.340	0.012	2.53	2.77	0.532	[0.12, 0.84]	0.000000
**CG right**	0.339	0.010	2.56	2.09	0.380	[0.05, 0.74]	0.000000
**CST left**	0.445	0.014	1.86	2.91	0.709	[0.23, 0.92]	0.000000
**CST right**	0.443	0.016	2.01	3.27	0.724	[0.27, 0.92]	0.000000
**FPT left**	0.410	0.016	2.84	3.20	0.557	[0.11, 0.86]	0.000000
**FPT right**	0.415	0.018	2.68	3.84	0.675	[0.18, 0.91]	0.000000
**FX left**	0.386	0.022	3.23	5.23	0.706	[0.43, 0.9]	0.000000
**FX right**	0.358	0.023	2.80	6.20	0.824	[0.6, 0.95]	0.000000
**ICP left**	0.331	0.007	1.49	1.83	0.558	[0.26, 0.84]	0.000088
**ICP right**	0.323	0.007	1.34	1.81	0.610	[0.3, 0.86]	0.000045
**IFO left**	0.348	0.010	1.09	2.91	0.875	[0.64, 0.97]	0.000000
**IFO right**	0.337	0.009	0.79	2.71	0.921	[0.79, 0.98]	0.000000
**ILF left**	0.355	0.013	1.31	3.73	0.887	[0.68, 0.97]	0.000000
**ILF right**	0.347	0.012	0.92	3.54	0.936	[0.84, 0.98]	0.000000
**MCP**	0.356	0.006	1.27	1.37	0.483	[0.18, 0.8]	0.000719
**MLF left**	0.349	0.007	0.68	2.06	0.898	[0.76, 0.97]	0.000000
**MLF right**	0.357	0.007	0.70	1.97	0.885	[0.72, 0.97]	0.000000
**OR left**	0.356	0.010	1.02	2.87	0.887	[0.66, 0.97]	0.000000
**OR right**	0.358	0.007	0.85	1.98	0.840	[0.64, 0.95]	0.000000
**POPT left**	0.398	0.013	1.09	3.17	0.893	[0.61, 0.97]	0.000000
**POPT right**	0.406	0.012	0.88	2.91	0.914	[0.72, 0.98]	0.000000
**SCP left**	0.374	0.010	1.22	2.43	0.792	[0.57, 0.93]	0.000000
**SCP right**	0.358	0.010	1.47	2.55	0.733	[0.47, 0.91]	0.000000
**SLF III left**	0.362	0.013	2.10	3.23	0.699	[0.28, 0.91]	0.000000
**SLF III right**	0.366	0.012	1.57	3.05	0.787	[0.39, 0.94]	0.000000
**SLF II left**	0.337	0.016	3.29	3.77	0.559	[0.12, 0.85]	0.000000
**SLF II right**	0.356	0.015	2.56	3.55	0.654	[0.2, 0.9]	0.000000

**SLF I left**	0.354	0.013	3.06	2.55	0.395	[0.05, 0.76]	0.000000
**SLF I right**	0.361	0.014	2.69	3.19	0.576	[0.14, 0.86]	0.000000
**ST FO left**	0.339	0.015	1.89	4.17	0.825	[0.62, 0.95]	0.000000
**ST FO right**	0.351	0.015	1.82	4.07	0.832	[0.62, 0.95]	0.000000
**ST OCC left**	0.362	0.010	0.94	2.67	0.889	[0.66, 0.97]	0.000000
**ST OCC right**	0.359	0.008	0.86	2.24	0.870	[0.68, 0.96]	0.000000
**ST PAR left**	0.373	0.010	0.87	2.69	0.902	[0.66, 0.97]	0.000000
**ST PAR right**	0.380	0.009	0.91	2.40	0.872	[0.64, 0.96]	0.000000
**ST POSTC left**	0.382	0.015	1.53	3.79	0.860	[0.48, 0.97]	0.000000
**ST POSTC right**	0.393	0.016	1.52	3.94	0.872	[0.57, 0.97]	0.000000
**ST PREC left**	0.392	0.013	1.91	2.99	0.708	[0.23, 0.92]	0.000000
**ST PREC right**	0.395	0.016	1.87	3.88	0.813	[0.39, 0.95]	0.000000
**ST PREF left**	0.341	0.013	2.84	2.85	0.493	[0.09, 0.82]	0.000000
**ST PREF right**	0.345	0.013	2.03	3.31	0.728	[0.25, 0.93]	0.000000
**ST PREM left**	0.354	0.019	3.65	4.52	0.604	[0.14, 0.88]	0.000000
**ST PREM right**	0.373	0.021	3.86	4.62	0.586	[0.15, 0.87]	0.000000
**STR left**	0.431	0.015	2.02	3.08	0.693	[0.27, 0.91]	0.000000
**STR right**	0.444	0.017	1.55	3.72	0.852	[0.43, 0.96]	0.000000
**T OCC left**	0.355	0.010	1.00	2.88	0.891	[0.68, 0.97]	0.000000
**T OCC right**	0.358	0.008	0.79	2.07	0.870	[0.69, 0.96]	0.000000
**T PAR left**	0.370	0.010	0.89	2.61	0.894	[0.65, 0.97]	0.000000
**T PAR right**	0.378	0.008	0.75	2.21	0.894	[0.7, 0.97]	0.000000
**T POSTC left**	0.384	0.014	1.40	3.49	0.860	[0.55, 0.96]	0.000000
**T POSTC right**	0.401	0.013	1.13	3.27	0.893	[0.68, 0.97]	0.000000
**T PREC left**	0.396	0.012	1.99	2.67	0.640	[0.16, 0.89]	0.000000
**T PREC right**	0.402	0.015	1.95	3.52	0.767	[0.29, 0.94]	0.000000
**T PREF left**	0.352	0.013	3.12	2.77	0.428	[0.07, 0.78]	0.000000
**T PREF right**	0.348	0.014	2.38	3.54	0.690	[0.19, 0.91]	0.000000
**T PREM left**	0.353	0.018	3.59	4.08	0.558	[0.12, 0.85]	0.000000
**T PREM right**	0.371	0.019	3.82	4.10	0.534	[0.1, 0.84]	0.000000
**UF left**	0.326	0.015	2.40	4.20	0.746	[0.37, 0.93]	0.000000
**UF right**	0.320	0.014	1.89	4.18	0.826	[0.58, 0.95]	0.000000

Table S7: Reproducibility and reliability of MD at the tract level

Tract	mean	std	CV_{ws}	CV_{bs}	ICC(A,1)	ICC 95% CI	p-value
**AF left**	7.37E-04	1.06E-05	1.20	1.01	0.384	[0.06, 0.74]	0.000002
**AF right**	7.33E-04	1.23E-05	1.01	1.48	0.674	[0.21, 0.9]	0.000000
**ATR left**	7.70E-04	1.32E-05	1.64	0.98	0.223	[0.01, 0.58]	0.000517
**ATR right**	7.68E-04	1.61E-05	1.37	1.79	0.618	[0.19, 0.88]	0.000000
**CA**	8.25E-04	2.63E-05	1.97	2.79	0.662	[0.21, 0.9]	0.000000
**CC 1**	7.98E-04	2.57E-05	1.75	2.95	0.727	[0.4, 0.91]	0.000000
**CC 2**	7.85E-04	1.54E-05	1.53	1.50	0.472	[0.09, 0.8]	0.000000
**CC 3**	7.96E-04	2.01E-05	1.91	1.98	0.503	[0.11, 0.82]	0.000000
**CC 4**	7.83E-04	1.89E-05	1.63	2.03	0.594	[0.22, 0.86]	0.000000
**CC 5**	8.10E-04	2.45E-05	1.69	2.75	0.720	[0.36, 0.91]	0.000000
**CC 6**	8.25E-04	1.87E-05	0.93	2.20	0.845	[0.64, 0.95]	0.000000
**CC 7**	8.58E-04	2.21E-05	1.06	2.50	0.847	[0.49, 0.96]	0.000000
**CC**	7.98E-04	1.45E-05	1.15	1.57	0.638	[0.23, 0.88]	0.000000
**CG left**	7.56E-04	1.55E-05	1.42	1.69	0.567	[0.16, 0.85]	0.000000
**CG right**	7.54E-04	1.53E-05	1.47	1.62	0.530	[0.13, 0.84]	0.000000
**CST left**	7.45E-04	1.59E-05	1.57	1.69	0.517	[0.13, 0.83]	0.000000
**CST right**	7.46E-04	1.51E-05	1.69	1.43	0.394	[0.06, 0.75]	0.000000
**FPT left**	7.80E-04	1.65E-05	1.67	1.59	0.451	[0.09, 0.79]	0.000000
**FPT right**	7.78E-04	1.88E-05	1.90	1.82	0.459	[0.08, 0.8]	0.000000
**FX left**	1.54E-03	1.43E-04	1.98	9.44	0.958	[0.89, 0.99]	0.000000
**FX right**	1.47E-03	1.41E-04	1.76	9.82	0.970	[0.92, 0.99]	0.000000
**ICP left**	7.31E-04	2.07E-05	1.72	2.50	0.656	[0.36, 0.88]	0.000001
**ICP right**	7.31E-04	1.87E-05	1.78	2.10	0.556	[0.21, 0.84]	0.000001
**IFO left**	7.93E-04	1.43E-05	1.10	1.58	0.662	[0.21, 0.9]	0.000000
**IFO right**	7.95E-04	1.95E-05	1.07	2.36	0.828	[0.39, 0.96]	0.000000
**ILF left**	8.11E-04	1.80E-05	1.18	2.05	0.744	[0.36, 0.92]	0.000000
**ILF right**	8.08E-04	2.21E-05	1.09	2.66	0.854	[0.48, 0.96]	0.000000
**MCP**	7.56E-04	1.85E-05	1.72	2.00	0.550	[0.2, 0.84]	0.000001
**MLF left**	7.94E-04	1.68E-05	1.01	2.00	0.789	[0.54, 0.93]	0.000000
**MLF right**	7.92E-04	1.79E-05	0.90	2.20	0.854	[0.66, 0.96]	0.000000
**OR left**	7.96E-04	1.58E-05	1.21	1.73	0.662	[0.22, 0.9]	0.000000
**OR right**	8.07E-04	2.23E-05	1.30	2.62	0.799	[0.44, 0.94]	0.000000
**POPT left**	8.08E-04	1.76E-05	1.29	1.93	0.678	[0.35, 0.89]	0.000000
**POPT right**	8.10E-04	1.95E-05	1.31	2.21	0.730	[0.42, 0.91]	0.000000
**SCP left**	7.42E-04	1.82E-05	1.66	2.05	0.586	[0.2, 0.86]	0.000000
**SCP right**	7.54E-04	1.94E-05	1.97	1.98	0.489	[0.09, 0.82]	0.000000
**SLF III left**	7.35E-04	1.07E-05	1.36	0.87	0.250	[0.02, 0.62]	0.000192
**SLF III right**	7.31E-04	1.23E-05	1.22	1.35	0.540	[0.11, 0.85]	0.000000
**SLF II left**	7.51E-04	1.14E-05	1.40	0.91	0.253	[0.02, 0.62]	0.000302
**SLF II right**	7.43E-04	1.43E-05	1.32	1.59	0.575	[0.17, 0.86]	0.000000

**SLF I left**	7.72E-04	2.07E-05	1.77	2.27	0.603	[0.23, 0.87]	0.000000
**SLF I right**	7.56E-04	1.76E-05	1.65	1.89	0.549	[0.17, 0.84]	0.000000
**ST FO left**	7.53E-04	1.47E-05	1.32	1.63	0.588	[0.22, 0.86]	0.000000
**ST FO right**	7.48E-04	1.83E-05	1.40	2.21	0.708	[0.28, 0.91]	0.000000
**ST OCC left**	8.01E-04	1.59E-05	1.21	1.75	0.664	[0.23, 0.9]	0.000000
**ST OCC right**	8.05E-04	2.34E-05	1.20	2.82	0.846	[0.49, 0.96]	0.000000
**ST PAR left**	7.88E-04	1.42E-05	1.11	1.58	0.650	[0.33, 0.88]	0.000000
**ST PAR right**	7.84E-04	1.72E-05	1.09	2.06	0.774	[0.51, 0.93]	0.000000
**ST POSTC left**	7.54E-04	1.37E-05	1.33	1.46	0.523	[0.16, 0.83]	0.000001
**ST POSTC right**	7.42E-04	1.58E-05	1.43	1.79	0.594	[0.21, 0.86]	0.000000
**ST PREC left**	7.20E-04	1.25E-05	1.45	1.23	0.387	[0.07, 0.74]	0.000005
**ST PREC right**	7.14E-04	1.31E-05	1.46	1.37	0.442	[0.09, 0.78]	0.000001
**ST PREF left**	7.46E-04	1.23E-05	1.50	1.02	0.287	[0.03, 0.66]	0.000007
**ST PREF right**	7.44E-04	1.48E-05	1.32	1.69	0.612	[0.15, 0.88]	0.000000
**ST PREM left**	7.31E-04	1.31E-05	1.70	1.03	0.242	[0.02, 0.61]	0.000009
**ST PREM right**	7.12E-04	1.35E-05	1.50	1.42	0.458	[0.07, 0.8]	0.000000
**STR left**	6.94E-04	1.30E-05	1.74	1.14	0.251	[0.02, 0.62]	0.001151
**STR right**	6.96E-04	1.53E-05	1.92	1.47	0.335	[0.05, 0.7]	0.000045
**T OCC left**	7.99E-04	1.52E-05	1.20	1.65	0.643	[0.2, 0.89]	0.000000
**T OCC right**	8.11E-04	2.27E-05	1.26	2.67	0.814	[0.47, 0.95]	0.000000
**T PAR left**	7.93E-04	1.41E-05	1.13	1.54	0.628	[0.31, 0.87]	0.000000
**T PAR right**	8.00E-04	1.99E-05	1.22	2.33	0.777	[0.53, 0.93]	0.000000
**T POSTC left**	7.60E-04	1.52E-05	1.42	1.63	0.551	[0.16, 0.84]	0.000000
**T POSTC right**	7.51E-04	1.91E-05	1.66	2.17	0.615	[0.22, 0.87]	0.000000
**T PREC left**	7.19E-04	1.26E-05	1.56	1.14	0.314	[0.04, 0.68]	0.000022
**T PREC right**	7.14E-04	1.31E-05	1.62	1.21	0.325	[0.04, 0.69]	0.000025
**T PREF left**	7.52E-04	1.23E-05	1.61	0.87	0.188	[0.0, 0.53]	0.000639
**T PREF right**	7.51E-04	1.50E-05	1.52	1.54	0.490	[0.1, 0.81]	0.000000
**T PREM left**	7.36E-04	1.40E-05	1.80	1.12	0.248	[0.02, 0.62]	0.000030
**T PREM right**	7.18E-04	1.45E-05	1.88	1.22	0.266	[0.02, 0.64]	0.000017
**UF left**	7.87E-04	1.83E-05	1.47	2.02	0.648	[0.16, 0.9]	0.000000
**UF right**	7.94E-04	1.94E-05	1.47	2.15	0.677	[0.17, 0.91]	0.000000

Additional results for tensor-based metrics using multi-shell data (Supplement to Section 3.3)

Tensor-based metrics were computed using a subset of the full multi-shell data (b=1000 s/mm^2), to fit the modelling assumptions for the tensor (as described in Section 2.4). While our primary findings of reproducibility and reliability of tensor-based metrics is derived from this limited dataset, for the purposes of transparency, we additionally provide reproducibility and reliability of tensor-based metrics using the full multi-shell data used for FOD modelling.

Table S8: Reproducibility and reliability of whole-brain averaged FA and MD using multi-shell data

Method	Metric	Mean	SD	CV_{ws} (%)	CV_{bs} (%)	ICC (95% CI)	ICC p-val
DTI (WM voxel mask)	FA	0.336	0.0068	1.05	1.88	0.760 (0.36, 0.93)	< 0.0001
	MD	7.1e-04	1.6e-05	0.86	2.20	0.865 (0.67, 0.96)	< 0.0001
DTI (FA mask)	FA	0.417	0.0089	1.19	1.95	0.727 (0.28, 0.92)	< 0.0001
	MD	6.2e-04	9.8e-06	0.90	1.42	0.706 (0.30, 0.91)	< 0.0001

Table S9: Summary metrics of reproducibility and reliability for tract-averaged FA and MD using multi-shell data

**Metric**	**CV_{ws} (%)**			**ICC(A,1)**		
	Mean	SD	Range	Mean	SD	Range
**FA**	1.49	0.70	[0.54 – 3.20]	0.785	0.137	[0.308 – 0.959]
**MD**	1.23	0.36	[0.69 – 3.19]	0.749	0.122	[0.378 – 0.971]

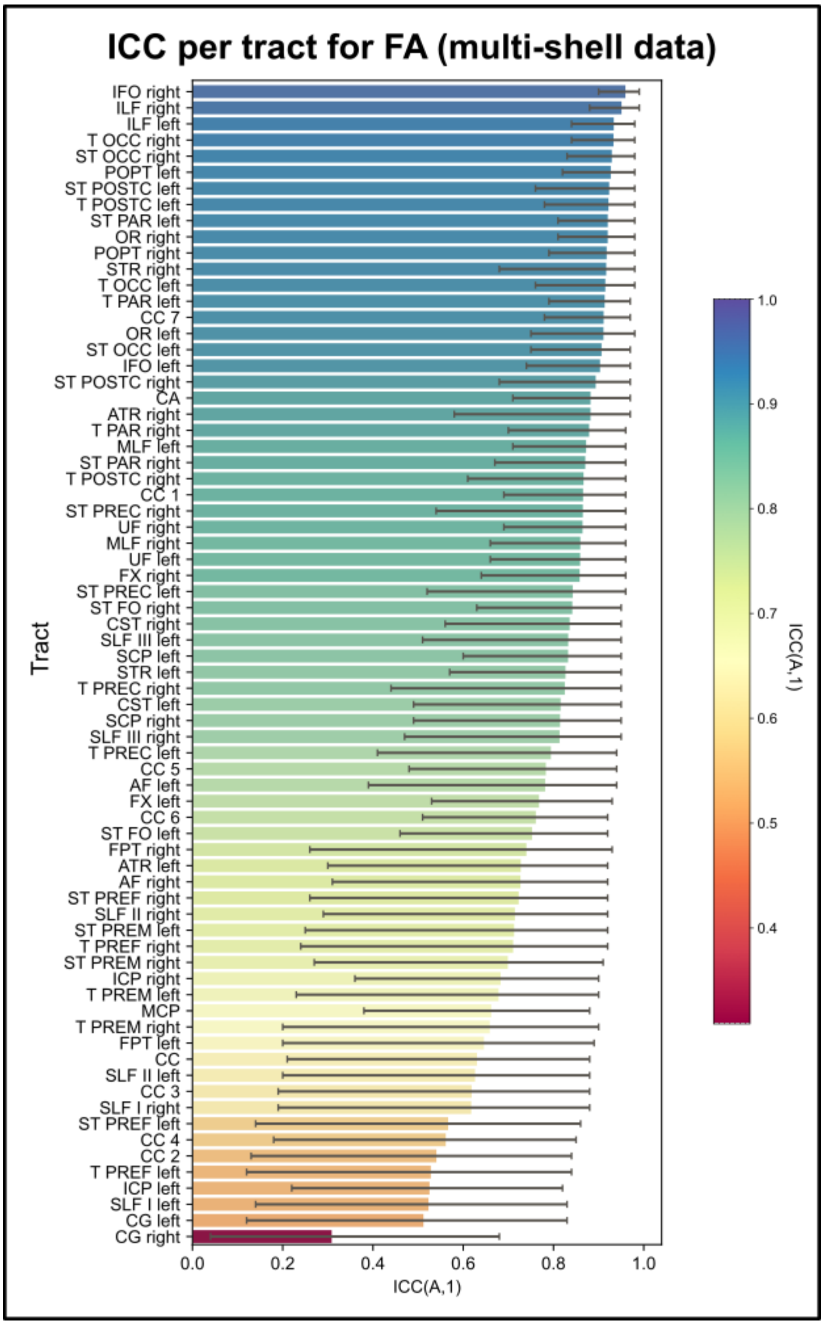

Supplementary Figure S9: Reliability of tract-averaged FA when using full multi-shell data.

Table S10: ICC of voxel-level FA and MD using multi-shell data

Method	Metric	Count	ICC(A,1)				
			Mean	Median	SD	Min	Max
DTI (WM voxel mask)	FA	431463	0.600	0.652	0.249	-0.3266	0.99478
	MD	431463	0.596	0.634	0.229	-0.3213	0.99746
DTI (FA mask)	FA	284920	0.601	0.654	0.250	-0.3266	0.99478
	MD	284920	0.567	0.599	0.231	-0.3213	0.99746

Table S11: CV_{ws} of voxel-level FA and MD using multi-shell data

Method	Metric	Count	CV_{ws}				
			Mean	Median	SD	Min	Max
DTI (WM voxel mask)	FA	431463	17.86	17.71	8.69	0.85	65.16
	MD	431463	6.52	4.60	5.40	1.18	130.89
DTI (FA mask)	FA	284920	15.29	14.23	8.16	0.85	63.40
	MD	284920	5.15	3.81	4.43	1.18	130.89

Additional results for different *b*-values (Supplement to Section 3.4)

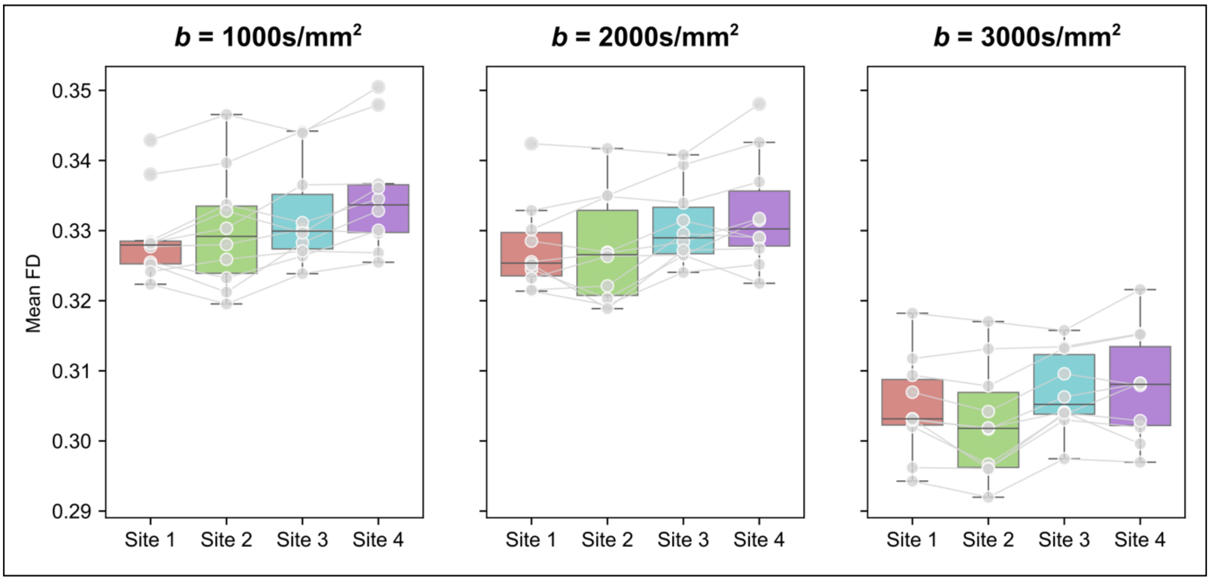

Supplementary Figure S10: Boxplots showing mean FD across the whole brain at different *b*-values. FD showed *b*-value dependence, but high and comparable reproducibility across all b-shells (see Supplementary Table S12).

Table S12: Reproducibility and reliability of whole-brain fibre density (FD) at different *b*-values

***b*-shell**	**Mean**	**SD**	**CV_{ws} (%)**	**CV_{bs} (%)**	**ICC (A,1)**	**ICC p-value**
b1000	0.332	0.0077	1.00	2.23	0.828 (0.5, 0.95)	<0.0001
b2000	0.329	0.0071	1.03	2.02	0.788 (0.47, 0.94)	<0.0001
b3000	0.306	0.0072	1.01	2.29	0.836 (0.54, 0.95)	<0.0001

Reported ICC values are for both two-way fixed effects, single measurement with absolute agreement (ICC(A,1)).

Table S13: Summary metrics of reproducibility and reliability for tract-averaged FD at different b-values

***b*-value**	**CV_{ws} (%)**			**ICC(A,1)**		
**(s/mm²)**	Mean	SD	Range	Mean	SD	Range
**1000**	1.78	0.47	[1.15 – 3.74]	0.791	0.118	[0.392 – 0.929]
**2000**	1.62	0.56	[0.99 – 3.53]	0.835	0.076	[0.511 – 0.937]
**3000**	1.38	0.63	[0.72 – 4.10]	0.891	0.066	[0.606 – 0.964]

Table S14: Summary metrics of reproducibility and reliability for fixel-wise FD at different b-values

b-value (s/mm^2)	CV_{ws} (%)			ICC(A,1)		
	Mean	SD	Range	Mean	SD	Range
**1000**	15.784	11.895	[1.33 – 200]	0.678	0.253	[-0.501 – 0.997]
**2000**	15.978	16.861	[1.20 – 200]	0.802	0.202	[-0.442 – 0.999]
**3000**	17.392	20.576	[1.04 – 200]	0.836	0.183	[-0.485 – 0.999]

Additional results for sample size estimates (Supplement to Section 3.5)

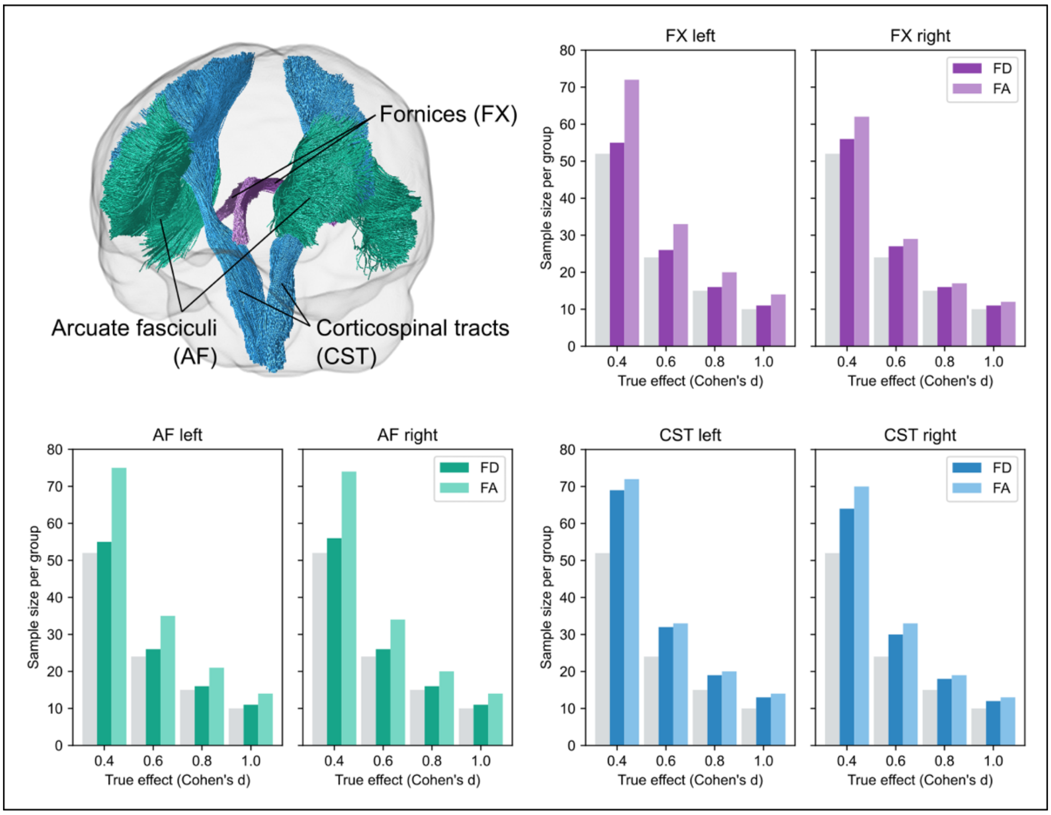

Supplementary Figure S11: Sample size estimates for tract-level analyses for paired t-tests. As with Figure 7 in the main paper, here we provide sample size estimates for tract-level analyses for 3 select tracts (fornix (FX), arcuate fasciculus (AF), and corticospinal tract (CST)), using ICC values computed from tract-level reproducibility analyses.
